# Clinical and cost-effectiveness of one-session treatment (OST) versus multisession cognitive–behavioural therapy (CBT) for specific phobias in children: protocol for a non-inferiority randomised controlled trial

**DOI:** 10.1136/bmjopen-2018-025031

**Published:** 2018-08-17

**Authors:** Barry D Wright, Cindy Cooper, Alexander J Scott, Lucy Tindall, Shehzad Ali, Penny Bee, Katie Biggs, Trilby Breckman, Thompson E Davis III, Lina Gega, Rebecca Julie Hargate, Ellen Lee, Karina Lovell, David Marshall, Dean McMillan, M Dawn Teare, Jonathan Wilson

**Affiliations:** 1 Child Oriented Mental Health Intervention Centre, IT Centre, Innovation Way, York, UK; 2 ScHARR, The University of Sheffield, Sheffield, UK; 3 Department of Health Sciences & Hull York Medical School, University of York, York, UK; 4 Division of Nursing, Midwifery and Social Work, University of Manchester, Manchester, UK; 5 Triumph Over Phobia (TOP UK), Bath, UK; 6 Psychological Services Center, Louisiana State University, Baton Rouge, Louisiana, USA; 7 Centre for Reviews and Dissemination, University of York, York, UK; 8 Research and Development, Norfolk and Suffolk NHS Foundation Trust, Norwich, UK

**Keywords:** phobia, children and adolescents, randomised controlled trial, one-session treatment, exposure therapy

## Abstract

**Introduction:**

Specific phobias (intense, enduring fears of an object or situation that lead to avoidance and severe distress) are highly prevalent among children and young people. Cognitive–behavioural therapy (CBT) is a well-established, effective intervention, but it can be time consuming and costly because it is routinely delivered over multiple sessions during several months. Alternative methods of treating severe and debilitating phobias in children are needed, like one-session treatment (OST), to reduce time and cost, and to prevent therapeutic drift and help children recover quickly. Our study explores whether (1) outcomes with OST are ‘no worse’ than outcomes with multisession CBT, (2) OST is acceptable to children, their parents and the practitioners who use it and (3) OST offers good value for money to the National Health Service (NHS) and to society.

**Method:**

A pragmatic, non-inferiority, randomised controlled trial will compare OST with multisession CBT-based therapy on their clinical and cost-effectiveness. The primary clinical outcome is a standardised behavioural task of approaching the feared stimulus at 6 months postrandomisation. The outcomes for the within-trial cost-effectiveness analysis are quality-adjusted life years based on EQ-5D-Y, and individual-level costs based of the intervention and use of health and social service care. A nested qualitative evaluation will explore children’s, parents’ and practitioners’ perceptions and experiences of OST. A total of 286 children, 7–16 years old, with DSM-IV diagnoses of specific phobia will be recruited via gatekeepers in the NHS, schools and voluntary youth services, and via public adverts.

**Ethics and dissemination:**

The trial received ethical approval from North East and York Research Ethics Committee (Reference: 17/NE/0012). Dissemination plans include publications in peer-reviewed journals, presentations in relevant research conferences, local research symposia and seminars for children and their families, and for professionals and service managers.

**Trial registration number:**

ISRCTN19883421; Pre-results.

**Strengths and limitations of this study**

- To our knowledge, this is the largest proposed treatment study and economic evaluation of interventions for childhood phobias to date.
- Interventions are delivered by multidisciplinary professionals working in multiple sectors including health, education and voluntary organisations.
- Children with all types of specific phobias and different learning abilities are included.
- There are no midpoint outcomes; therefore, the study cannot tell whether children improve with one-session treatment quicker than with multisession therapy or how the two interventions may work in different ways to change outcomes (mechanisms of action). However, the study includes longer term outcomes which are useful in evaluating sustained effect, if any, of the intervention.

## Introduction

A specific phobia is an intense, enduring fear of an identifiable object or situation that leads to distress and avoidance.^1^ It is estimated that between 5% and 10% of children have a specific phobia severe enough to impact on their everyday functioning,^2^ but fewer than 10% seek treatment, therefore, the average duration of phobias is 20 years.^3^ The psychological, developmental and medical impact of these phobias is significant, with higher rates of health service usage than most other anxiety disorders.^4^ Phobias in children can result in considerable academic difficulties,^5^ personal distress^6^ and interference in day-to-day activities.^7^ Cognitive– behavioural therapy (CBT) is the dominant therapeutic intervention for specific phobias^8 9^ within health services in the UK. Robust evidence supports the efficacy of CBT for anxiety disorders in general,^10–12^ and for specific phobias in particular; however, CBT can be time consuming, costly and difficult to access because it is delivered over multiple sessions during several weeks or months by therapists that have received lengthy training.^13–16^

Alternative methods of CBT delivery, like one-session treatment (OST),^17^ can be used to reduce demands on therapist time and associated costs, prevent therapeutic drift, and ultimately help children recover quicker.^18 19^ OST is a variant of CBT, delivered in a single 3-hour session. A central feature is graded exposure where the subject comes into proximity with the phobic stimulus in a series of graduated steps (from the easiest to the most difficult). For example, graded exposure for a phobia of dogs might first involve looking at pictures of dogs, followed by videos of dogs, then by observing dogs in a local park at a distance and finally by interacting with dogs first hand. OST has been shown to be clinically effective in children.^20–22^ For example, Ollendick *et al* 20 randomised 196 children (ages 7–16) with phobias to one of three groups: (1) OST, (2) an education support group or (3) a wait-list control group. OST was superior to both comparators as measured by improved phobia severity, percentage of children who were diagnosis free, and child-related and parent-rated anxiety and treatment satisfaction postintervention and at 6 months follow-up. However, to our knowledge, OST has not been compared with the current ‘gold-standard’ approach to specific phobias (ie, CBT), and has not been trialled in the UK within the National Health Service (NHS).

The present research is a non-inferiority, parallel group randomised controlled trial comparing the clinical and cost-effectiveness of OST versus CBT for specific phobias in children and young people ages 7–16 years old. The primary aim is to test whether OST is non-inferior (ie, produces similar clinical benefits) to multisession CBT in helping children with their specific phobia(s), and the wider impact their phobia(s) have on mental health and quality of life. A nested qualitative evaluation will explore whether OST is acceptable to children, their parents and the practitioners who deliver OST. Finally, an economic evaluation will examine whether OST offers good value for money to the NHS and society compared with multisession CBT.

## Methods and analysis

### Participants

The present research will recruit children and young people aged 7–16 years old with one or more specific phobias, as defined by the Diagnostic and Statistical Manual of Mental Disorders, Fifth Edition 10 Add Department of Rheumatology.^1^ The DSM-IV criteria are: (1) marked and out-of-proportion fear to a specific object or situation, (2) exposure to the feared object or situation provokes immediate anxiety, (3) the phobic situation is avoided where possible, (4) the avoidance or distress associated with the phobia interferes with the person’s routine or functioning (eg, learning, sleep, social activities) and (5) the phobic avoidance or distress is present for 6 months or more. We include all types of phobias where we can introduce or safely simulate the stimulus for the purpose of exposure therapy. We exclude children who have conditions that are often referred to as ‘phobias’ but are not specific phobias (eg, social or separation anxiety referred to in some circumstances as ‘school phobia’) or when children need a different intervention as a priority (eg, for psychosis or self-harm).

### Design

This is a non-inferiority randomised controlled trial that compares clinical outcomes of OST with those of multisession CBT at 6 months postrandomisation. Nested within this trial is an economic evaluation of the cost-effectiveness of OST against CBT for outcomes and costs over 6 months. Interviews evaluate perceptions and experiences of OST by children, parents and practitioners also at 6-month follow-up. An internal 9-month pilot determines feasibility of recruitment and retention, and monitors the occurrence of any adverse events.

### Recruitment

Our recruitment strategies follow routine care pathways for children with specific phobias. These pathways differ across our participating sites, which include NHS Trusts, Local Authorities and voluntary agencies across England. We have mapped the care pathways in each geographical area and we promote the study within teams of professionals who routinely receive referrals of children with specific phobias, such as Child and Adolescent Mental Health Services (CAMHS), Children’s Hospitals, youth services and schools. We also draw attention of the study to potential participants through public adverts, for example, in local newspapers and posters in general practitioner (GP) surgeries.

### Randomisation

Following written consent and completion of the baseline measures, random allocation to treatment groups is carried out remotely through a secure web-based programme designed by the Sheffield Clinical Trials Research Unit (CTRU). The randomisation allocation ratio is 1:1 to facilitate equal sample sizes across both the OST and CBT groups. Randomisation is stratified according to two age groups (7–11 years old vs 12–16 years old) and two phobia severity groups (mild to moderate vs severe); we use random permuted blocks of variable size to ensure enough participants are allocated evenly to each arm of the trial within each stratum.

### Blinding

Complete concealment of allocation, ‘blinding’, from both participants and researchers in studies of psychological interventions is often extremely difficult, and, at times, impossible.^23–26^ Our study takes a number of steps to facilitate blind assessment of outcomes as a way of reducing bias. First, the research assistants collecting baseline and follow-up outcomes are independent of the practitioners delivering the interventions. Second, the research assistants are not informed of the participants’ group allocation and are not involved in the therapy sessions. Participants are explicitly reminded not to disclose to the research assistant their allocated group. In the event of these procedures being compromised (eg, a research assistant learning the participant’s allocated group), we record this and arrange for a different outcome assessor in the future wherever possible. The trial statisticians and health economists are blind to treatment allocation while the trial is ongoing.

### Interventions

#### One-session treatment

OST is a variant of CBT, but rather than being delivered in weekly 45–60 min sessions as in conventional CBT, OST is delivered in a longer single session (approximately 3 hours). Prior to the single session, a 1-hour assessment/planning session takes place during which the practitioner carries out an assessment in order to plan the treatment session. This involves:

1. Determining the nature, history and associations of the phobia.
2. Determining any maintaining factors for the phobia (eg, what the child avoids, what safety behaviours she/he engages in, how friends and family may collude with the phobia).
3. Collecting information about the child’s cognitions including catastrophic thoughts (eg, what do you think may happen if…, what goes through your mind when you…).
4. Generating a fear hierarchy (ie, a ‘ladder’ of situations or objects that the child avoids because of their phobia, in the order of the fear or anxiety evoked, starting from the least frightening).
5. Explaining what is involved in treatment and that the exposure tasks are graded and negotiated in every step of the way and that nothing will ever happen without the child’s permission.

The single session combines graded exposure, participant modelling, reinforcement and cognitive challenges. The main principle of graded exposure is that the child gradually confronts the situations from their fear hierarchy and remains in each situation until their anxiety/fear subsides at least by 50%. In participant modelling, the therapist first demonstrates to the child how to interact with the phobic object and then helps the child gradually approach the phobic object by joint approaches with the therapist gradually fading involvement. Reinforcement from the therapist may take the form of social praise, encouragement. Finally, the therapist uses the exposure tasks as means for actively eliciting, challenging and testing negative cognitions or catastrophic thoughts associated with the feared situation and by promoting positive cognitions in phobic situations. It is important that the child maximises, maintains and generalises their gains from graded exposure by practising self-directed and parent/carer supported exposure tasks at home for the ensuing weeks. Maintenance tasks include a commitment to refrain from avoiding or escaping from feared situations and to deliberately engineer or enter a feared situation regularly.

#### Cognitive–behavioural therapy

CBT helps people to change unhelpful beliefs and behaviours arising in feared situations, such as catastrophic thinking and safety behaviours. Interventions based on CBT are the dominant model of treatment for specific phobias, and are supported by a robust literature attesting to its efficacy.^8–12^ CBT interventions are often delivered in 45–60 min sessions every week or fortnight over several weeks or months. Each CBT session takes the form of structured discussions with a therapist, has a specific agenda and sets homework tasks for the child or young person between sessions. CBT for children and young people with specific phobias aims to help the child/young person to: (1) recognise anxious feelings and bodily reactions to anxiety; (2) gradually confront their feared situations until their anxiety subsides; (3) capture and challenge anxious thoughts when faced with a phobic situation or object; (4) challenge avoidance behaviours, or behaviours that maintain or reinforce the phobia and (5) develop coping strategies and anxiety management techniques if distress and physical responses to anxiety are overwhelming. There is no recommended number of CBT sessions for specific phobias but it can vary between 4 and 20 sessions.

### Fidelity to the interventions

Practitioners responsible for specific phobia treatment across the recruiting centres will be trained in OST during a day’s workshop, with senior practitioners (eg, trained therapists) attending a modified half-day training on OST and a half-day training on supervising and training others in OST to be able to offer continuous support and supervision to junior colleagues. OST and CBT sessions are audio recorded whenever possible with consent from the child and parent. A random selection of 15% of participants at each site (or the nearest whole number) has one of their sessions (whether OST or CBT) reviewed by a supervisor at quarterly intervals. Supervisors score the recorded sessions to assess therapist fidelity to OST and CBT using the OST Rating Scale 27 and the CBT Scale for Children and Young People,^28^ respectively. This is to make sure that CBT principles are applied in both treatment arms. These are used in supervision to improve fidelity to the intervention and address training needs for the therapists as per usual practice.

### Outcome measures

We are collecting demographic information (eg, age, gender, etc) from all children/young people and their parents. Children and parents are also asked if they have a treatment preference at baseline, although this does not affect their randomisation in any way. Participants complete the following measures at baseline and then at 6 months follow-up.

#### Behavioural avoidance task

The primary outcome is the behavioural avoidance task (BAT) at 6 months postrandomisation. The BAT is a widely used measure of treatment outcomes for phobias in children.^6 21^ The BATs for the Alleviating Specific Phobias Experienced by Children Trial (ASPECT) trial are standardised for each stimulus and have been found to have good test–retest reliability^20 29^ and strong correlation with other outcome measures of anxiety and phobia.^30 31^ During a BAT, the child gradually confronts their phobic stimulus over 10 predefined steps of increasing difficulty; the child does not have to complete all the steps. For example, a child with spider phobia may start the BAT at step 0, standing outside of a room that has a spider contained in a box. Subsequent steps the child may take are: opening the door (step 1), passing through the doorway (step 2), through to holding the spider for 20 s (step 10). The number of steps the participant takes is the main unit of measurement recorded for analysis. The BAT also includes a measure of subjective units of distress whereby the child reports their level of fear at the start of the BAT, and at the last step completed (ranging from 0=no fear at all to 8=extreme fear).

#### The anxiety disorder interview schedule for children and parents

Anxiety disorder interview schedule (ADIS)^32^ is a well-established semistructured interview^3 32 33^ that obtains information from both the child and their parent/carer about the type of specific phobias experienced (eg, animal/insect, small spaces, blood/injection phobias, etc), the degree of associated fear (rated from 0=not at all to 8=very, very much), whether the phobia causes avoidance (rated as ‘yes’ or ‘no’) and interference with daily life (rated from 0=not at all to 8=very, very much).

#### Child Anxiety Impact Scale for children and parents

The Child Anxiety Impact Scale for children and parent (CAIS-C/P)^34^ collects information as to what extent feeling ‘nervous, anxious or afraid’ impacts on the daily life of the child. The child and the parent independently rate, on a 4-point scale ranging from 0=not at all to 4=very much, 27 statements grouped in 3 subdomains: school activities (eg, getting to school on time in the morning), social life (eg, making new friends) and home/family life (eg, getting ready for bed at night).

#### The Revised Children’s Anxiety and Depression Scale

The Revised Children’s Anxiety and Depression Scale-C/P (RCADS-C/P)^35–38^ comprises 47 items to capture separation anxiety disorder, social phobia, generalised anxiety disorder, panic disorder, obsessive compulsive disorder and major depressive disorder. Children state the frequency of which statements such as ‘I worry about things’, I have trouble sleeping’ and ‘I feel I will make a fool of myself in front of people’ relate to them on a 4-point scale: 0=never, through to 1=sometimes, 2=often and 3=always.

#### EQ-5D-Y

The EQ-5D-Y, originally developed by the EuroQoL group,^39–41^ is a widely used measure of health-related quality of life in children and young people. Children and young people are able to classify their own health on a 3-point scale, 1=no problems, 2=some problems and 3=a lot of problems, over five dimensions: mobility (walking about), looking after myself, doing unusual activities, having pain or discomfort and feeling worried, sad or unhappy. Additionally, the EQ-5D-Y includes a Visual Analogue Scale where participants can indicate their overall health status from 0 (worst imaginable state) to 100 (best imaginable state). All questions refer to the participant’s health state ‘today’.

#### Child Health Utility 9D

The Child Health Utility 9D (CHU-9D) is a health-related quality of life measure validated for children and young people.^42 43^ Participants select a sentence from a possible five to describe how they feel today in relation to a number of constructs (ie, sadness, tiredness, pain, etc). For example, participants are asked to select one of five statements that best reflects how worried they feel ranging from ‘I don’t feel worried today’ to ‘I feel very worried today’.

#### Goal-based outcome measure

A goal-based outcome measure is a method of comparing how far a participant feels they have moved towards reaching a specific goal they have set before the intervention has begun. A goal-based outcome measure based on recent guidelines^44^ will be used to set up to three goals at baseline, with progress towards meeting these goals rated on a 10-point scale at the 6-month follow-up point. Progress can range from 0 (ie, goal not met), to 5 (ie, halfway to reaching the goal), through to 10 (ie, goal reached). For example, a participant with a dog phobia may set as their goal that they would like to be able to spend teatime at their grandmother’s house where there is a dog.

#### Resource Utilisation Questionnaire

We developed a bespoke questionnaire to assess use of healthcare and other resources, based on a previous Resource Utilisation Questionnaire by Barrett and colleagues^45^ and its adaptations for younger populations by members of our research team^45–47^ The questionnaire is completed by the parent and collects data on resource and service utilisation relating to the child over the previous 6 months on: general health and community service use (ie, appointments with GP’s, nurses, social services and educational services); mental health service use (ie, appointments with a psychiatrist, psychotherapists, psychologists, CAMHS therapists and other forms of mental health support); hospital-based services (ie, visits to A&E, urgent care centres and hospital stays); days missed from school by the child; days missed by parents/carers from work or studies.

### Statistical analysis

#### Sample size

The proposed sample size and non-inferiority margin are based on two separate Cochrane reviews looking at the effects of psychotherapy for those experiencing anxiety. First, Wolitzky-Taylor *et al* 47 conducted a review on studies that used both observer-rated BAT-like assessments and self-report questionnaires on adults with specific phobias and reported an overall, large effect size of d=0.81. However, as the treatment may have a different effect on children, we also examined the review of Reynolds *et al*.^48^ This review was conducted on studies of children with specific phobias but used self-report questionnaires rather than observer-rated BATs. The review reported a large effect size (d=0.85) for multisession CBT. Prior meta-analyses suggest that a standardised mean difference of around 0.8 on the BAT scale is clinically important. Therefore, we set the non-inferiority margin to be half of this at 0.4.^49^ Assuming a correlation of 0.5 between baseline and final BAT measure, we would require 200 participants (100 in each arm) to have 90% power with a 2.5% one-sided significance level to demonstrate non-inferiority of OST compared with CBT. The therapy is delivered by therapists who will see approximately 15 patients each and we anticipate a weak therapist effect (intraclass correlation coefficient=0.01). This clustering will lead to a design effect of 1.14 which increases the number required per arm to 114. We further assume a 20% drop-out rate suggesting that total number of 286 children and young people (143 per arm) will need to be recruited to the study to demonstrate non-inferiority of OST compared with CBT.

#### Data analysis

As this trial follows a randomised, parallel group, non-inferiority design, data will be analysed and reported according to both Consolidated Standards Of Reporting Trials (CONSORT) guidelines^50^ and the non-inferiority trials CONSORT extension.^51^ Baseline data, including demographic characteristics (eg, age, gender) and clinical measures (eg, BAT, RCADS), will be reported between the two randomised groups. Jones *et al* 49 recommend both per-protocol and intention-to-treat (ITT) analyses for non-inferiority designs. For an equivalence or non-inferiority trial, as opposed to a comparative trial, ITT analyses are not considered conservative, as any blurring of the difference between the treatment groups increases the chance of declaring equivalence. We follow this recommendation with the refinement that the main analysis of the primary outcome will be per protocol (or completers only) with a sensitivity analysis on the ITT population.^49^ We will require both the per-protocol and ITT analyses to demonstrate conclusive evidence to declare that OST is ‘non-inferior’ to multisession CBT. If the results of the analysis are discrepant (eg, the ITT rejects the null of inferiority but the per-protocol analysis does not or vice versa) then we will report the conflicting results from both analyses highlighting the inconclusive nature of the results. For the sake of the analysis, a participant in the OST group is defined as per protocol if they attend both the initial functional assessment and the rapid exposure therapy session. A participant in the CBT group is defined as per protocol if they attend at least four CBT sessions.

The primary outcome (mean BAT score at 6 months) and the secondary outcomes (ADIS, CAIS, EQ-5D-Y, CHU-9D, RCADS and goal-based outcome scores) will be compared between groups using mixed-effects linear regression with exchangeable correlation to allow for the clustering of outcomes within therapist. The analysis will be conducted controlling for baseline BAT score, site and stratifying variables (age group and baseline phobia severity). The null hypothesis of inferiority will be rejected if the two-sided 95% CI for the difference is wholly below 0.4 (the range of clinical non-inferiority).

We anticipate some drop-out/attrition, so case missing data may be an issue. Case and item missing data will be examined and multiple imputation methods will be used to reduce bias due to any missing responses in both the per-protocol and the ITT analyses. Where appropriate, modelling methods that generate robust SEs in the presence of missing data will be considered.

### Economic evaluation

We are conducting an economic evaluation from the UK personal and social services perspective. This will take the form of a within-trial cost–utility analysis to determine the incremental cost per unit of quality-adjusted life years (QALYs) of OST compared with CBT in children with specific phobias. Costs will be calculated based on: (1) resources required to deliver the intervention and (2) individual-level use of health and social services by children and absenteeism from school for children or from work because of childcare for parents over the study period. Resource use will be multiplied by unit costs to arrive at total cost in each arm. QALYs will be calculated by measuring health-related quality of life using the self-reported EQ-5D-Y over the study period.

The resources required to deliver the OST intervention will be calculated using bottom–up estimation of the time spent by professionals as well as other resources used (including phobic stimulus acquisition, ie, animal hire). Individual-level service use data will be based on self-reported use of primary and secondary healthcare as well as social care using a standardised Resource Utilisation Questionnaire. Unit costs of health and social service use will be obtained from the UK national database of reference costs department of health,^52^ and the Unit Costs of Health and Social Care report produced by the Personal Social Services Research Unit.^53^ The cost of medication will be based on the most recent version of the British National Formulary Royal Pharmaceutical Society of Great Britain.^54^

Individual-level responses on the EQ-5D-Y will be used to estimate health-related quality of life based on a UK population valuation set. Subsequently, an area under the curve approach will be used to calculate QALYs for each child. QALY is a utility-based measure, that is, it measures each person’s health state in terms of quality of life dimensions and then weights it on the value or utility of the health state based on UK population preferences.^55^

The primary economic analysis will be cost–utility analysis conducted over the trial follow-up period (6 months). Total costs, including the intervention cost and service utilisation costs, and QALYs, will be compared between the two interventions. Unadjusted costs and outcomes will be presented in a descriptive analysis using parametric and non-parametric tests. The statistical analysis will compare mean costs and QALYs using a regression model. The regression analysis will control for baseline differences in utility.^56^ The specification of the model will follow the approach recommended by Glick *et al* 57 which considers the distribution of the dependent variable as well as any correlation between the cost and QALY outcomes. The regression coefficient on treatment will then represent the difference in mean cost and mean QALYs between groups. A bootstrap method will be used to produce CIs around the cost and QALY differences due to the likely skewness in the distribution of regression residuals.^58^ To present this in the UK decision-making context, the results will be in the conventional form of a cost-effectiveness acceptability curve (CEAC). CEAC presents the probability of the intervention being cost-effective over a range of willingness-to-pay (WTP) thresholds per QALY.^59^ The higher the probability, the more likely it is that the treatment is cost-effective at the particular WTP threshold.

The following sensitivity analyses will be conducted; (1) a cost–utility analysis using the CHU-9D instrument instead of EQ-5D; (2) a cost-effectiveness analysis using a phobia-specific measure instead of a utility-based measure and (3) cost–utility analysis from a health services perspective.

### Nested qualitative evaluation

We will invite a subsample of trial participants to participate in individual semistructured interviews and use maximum variation sampling to ensure a spread of participants differing in age, gender, socioeconomic background and type of phobia. We will obtain written consent (and assent where appropriate) to interview a sample of (1) children receiving OST, (2) their parents/guardians and (3) practitioners delivering OST across all study sites. With parental consent we will recruit and interview parents and children separately. Joint parent–child interviews will be included where participants prefer. The final sample size for the interviews in each participant group will be determined by data saturation, that is, the point where no new themes, ideas and/or concepts emerge. Based on a previous nested qualitative evaluation of patient acceptability of brief psychological interventions,^60^ we estimate that we will need to complete a maximum of 30 interviews with parents, 25 with children and 15 with practitioners.

Interviews with children and their parents will be conducted after participants have completed the final outcome measures at the 6-month follow-up point. Interviews for parents will focus on phobia experiences, personal and family impact, perceived treatment need, treatment expectations and treatment acceptability (eg, content, delivery mode, format, setting and facilitation). Child interviews will focus on the same topics, adapted for age and developmental maturity. Face-to-face interviews with children will draw on the principles of ‘draw and write’ techniques, whereby children will be offered an opportunity to draw a picture relating to their experiences as a prompt to initiate more in-depth discussion.^61 62^ Child interviews will last a maximum of 30 min and parent interviews a maximum of 60 min, with total time guided by the interviewees. Clinician interviews will last for a maximum of 60 min and focus on their experiences and views of delivering OST, the individual and organisational support required and the perceived suitability OST for their patient group.

Interviews with parents and older children (13 years plus) will be conducted face to face in treatment settings or at participants’ homes or via the telephone, depending on participant preference. Interviews with younger children (12 years and under) will be conducted face to face in treatment settings or at participants’ homes, depending on participant preference. Practitioner interviews will take place face to face in their workplace or over the telephone once their own involvement in the trial is complete.

All interviews will be digitally recorded and transcribed verbatim. Analysis will follow a qualitative framework approach,^63^ a widely used method of analysing primary qualitative data pertaining to healthcare practices with policy relevance.^64^ Framework analysis permits both deductive and inductive coding, enabling potentially important themes or concepts that have been identified a priori to be combined with additional themes emerging de novo.

Data coding will be undertaken independently by two trained researchers. We will additionally train a patient and public involvement (PPI) representative to code alongside these researchers to ensure coding takes account of potential differences in perspective. Coders will meet regularly to develop a shared coding manual and to ensure that all emerging codes remain grounded in original data. An Excel spread sheet will be developed which will incorporate preliminary framework themes as column headings and the demographic information related to participants who provided data under each theme. As the constant comparison of new data occurs and the coding team’s understandings of the themes under consideration develop, the framework will be amended and reshaped to enable the introduction of new codes and/or the deletion of redundant, similar or otherwise compromised codes. In this way, a final framework will be achieved that is considered representative of the entire dataset. We will code data from each stakeholder group (children, parents/guardians and practitioners) separately before comparing and contrasting data findings across groups. The final coding manuals for each participant group will be presented with example entries, to the project’s advisory committees to confirm validity, coherence and conceptual relevance.

## Public and patient involvement

The research protocol was developed in partnership with service user groups and was reviewed in terms of both feasibility and relevance. In addition to this, the views of relevant charitable organisations were sought and a representative from the Triumph Over Phobia UK support group is a coapplicant on this application. We have consulted with PPI representatives about their views on the full research proposal and how best to involve patients throughout the proposed project. In particular, we have discussed comments after the first round application with representatives of Triumph Over Phobia. This includes issues around the non-inferiority design, how to measure fidelity and contamination. We have also engaged the York Youth Council, who have commented on this application and discussed the methodology of the trial and the potential impact of the therapy on the community. We see PPI as central to our research. A representative from Triumph Over Phobia is on our trial management group (TMG) and actively involved in the planning and delivery of this research. Applicants KL and PB are named applicants (PI and Co-PI, respectively) on a National Institute for Health Research (NIHR)-funded programme grant seeking to improve user involvement in mental healthcare planning. This programme has been recognised by the former Mental Health Research Network (MHRN) for exemplary carer involvement. Applicant PB has substantial experience of training users and carers in research methodologies, interviewing skills and qualitative data analysis. We recognise the need for independent qualitative data analysis, and will train a service user researcher. The service user representative will be reimbursed for their time, commensurate with current INVOLVE guidelines.

## Ethics and dissemination

### Obtaining informed consent from participants

As all participants in this study are aged 16 or under, consent is required from both a person with parental responsibility, and the participant themselves if they are deemed competent to do so. Where a child is not deemed competent to give full informed consent, we take parental consent and seek assent from the child. Prior to the consent meeting, the parents and the children receive information about the study and have the opportunity to discuss any questions or concerns with members of the research team. Two age-specific versions of the child information sheets are available: one for those aged 7–11 years and one for those aged 12–16 years. All information leaflets and consent forms are codeveloped by the research team and PPI representatives to ensure acceptability among participants. If a participant wishes to withdraw from their allocated intervention, we check whether they are still agreeable to participate in the planned follow-up assessment at 6 months, so that their outcomes can be included within our ITT analysis.

### Data management

Trial data are extracted from source documents and entered onto the CTRU’s in-house data management system (Prospect). Prospect stores data in a PostgreSQL database hosted by the University of Sheffield. The database uses industry standard techniques to provide security, including password authentication and encryption using Secure Sockets Layer (SSL) /Transport Layer Security (TLS). Access to Prospect is controlled by user names and encrypted passwords. A comprehensive privilege management feature ensures only the minimum amount of data required is available to each individual to complete their tasks. The system has a full electronic audit trail and is regularly backed up. Access to data is restricted to users with appropriate privileges only. All data are collected and retained in accordance with the Data Protection Act 1998 and Sheffield CTRU standard operating procedures (SOPs).

### Research governance and conduct of the trial

Trial monitoring procedures and site monitoring are undertaken at a level appropriate to a risk assessment performed by the Trial Sponsor (Leeds and York Partnership NHS Foundation Trust, 2150 Century Way, Thorpe Park, Leeds, LS15 8ZB) and the Sheffield CTRU. Three committees govern the conduct of this study, in accordance with Sheffield CTRU’s SOPs: a trial steering committee (TSC), an independent data monitoring and ethics committee (DMEC) and a TMG.

The TSC consists of an independent chair, an independent subject specialist, an independent clinical academic, an independent statistician and a patient representative. The TSC meets approximately every 6 months from the start of the trial. The DMEC consists of an independent chair, an independent statistician and an independent ethics specialist experienced in research with children and families. The TMG comprises the coapplicants and the two trial managers who are jointly supervised by the Chief Investogator (CI), the director of the Sheffield CTRU and a lead trial manager in the CTRU. Meeting attendance of the coapplicants depends on each meeting’s agenda and relevance to their role.

### Adverse events

An adverse event monitoring form is used to record any untoward occurrence affecting the participant after each therapy session by the therapist and at follow-up by the research assistant. Such an event can be directly related, possibly related or unrelated to the intervention. An occurrence is recorded if it is suspected to be related to the intervention or an aspect of the research procedures; the therapist assesses relatedness and research assistants seek advice from the site principal investigator. The occurrence of adverse events during the trial is monitored by the trial oversight and management groups, particularly the DMEC and the TSC. All adverse events are assessed for seriousness, and will be recorded as a serious adverse event) if they are life threatening, or require hospitalisation or prolongation of existing inpatient stay, or result in persistent or significant disability or incapacity or death.

No pharmaceutical compounds or medical devices are used in this trial, therefore, clinical trials authorisation is not required.

### Dissemination of the research findings

We will publish the study’s findings in peer-reviewed academic journals and present at local, national and international conferences where possible. Furthermore, we will publish a short summary of the results on the ASPECT study website that can be accessed by all trial participants as well as relevant interest groups, including patient groups. Finally, towards the end of the trial, our PPI representatives will organise a meeting with stakeholders including parents and professionals working with young people with anxiety disorders to specifically discuss the dissemination of the study findings and put together a dissemination plan.

### Trial status

Protocol V.3, 24/04/17. Recruitment to the trial began in June 2017 and is estimated to be completed in July 2019, with the final follow-up assessments completed in March 2020.

## Supplementary Material

Reviewer comments

Author's manuscript
