# Supplementary material for: Clinical and cost-effectiveness of one-session treatment (OST) versus multisession cognitive–behavioural therapy (CBT) for specific phobias in children: protocol for a non-inferiority randomised controlled trial
Source: BMJ Open. 2018 Aug 17;8(8):e025031. doi: 10.1136/bmjopen-2018-025031 (PMC6104754; doi:10.1136/bmjopen-2018-025031)
Supplement: Author's manuscript [file bmjopen-2018-025031.draft_revisions.pdf]

BMJ Open is committed to open peer review. As part of this commitment we make the peer review history of every article we publish publicly available.

When an article is published we post the peer reviewers' comments and the authors' responses online. We also post the versions of the paper that were used during peer review. These are the versions that the peer review comments apply to.

The versions of the paper that follow are the versions that were submitted during the peer review process. They are not the versions of record or the final published versions. They should not be cited or distributed as the published version of this manuscript.

BMJ Open is an open access journal and the full, final, typeset and author-corrected version of record of the manuscript is available on our site with no access controls, subscription charges or pay-per-view fees (http://bmjopen.bmj.com).

If you have any questions on BMJ Open's open peer review process please email info.bmjopen@bmj.com

BMJ Open

Clinical and Cost-effectiveness of One Session Treatment (OST) vs. Multi-session Cognitive Behaviour Therapy (CBT) for Specific Phobias in Children: Protocol for a Non-inferiority Randomised Controlled Trial

Journal:	*BMJ Open*
Manuscript ID	bmjopen-2018-025031
Article Type:	Protocol
Date Submitted by the Author:	29-Jun-2018

Complete List of Authors:	Wright, Barry; Lime Trees CAMHS,<br>Scott, Alex; Sheffield University, School of Health and Related Research<br>Cooper, Cindy<br>Marshall, David; Lime Trees CAMHS , Research Team<br>tindall, Lucy<br>Davis III, Thompson<br>Teare, M; University of Sheffield, School of Health and Related Research<br>Lee, Ellen; University of Sheffield, Clinical Trials Research Unit<br>Lovell, Karina; The University of Manchester, The School of Nursing, Midwifery and Social Work<br>Bee, Penny; University of Manchester,<br>Gega, L; University of York<br>Hargate, Rebecca; Lime Trees CAMHS Centre, Research<br>Wilson, Jon<br>Ali, Shehzad; University of York, Department of Health Sciences<br>Biggs, Katie; University of Sheffield, Clinical Trials Unit<br>McMillan, Dean; University of York, Department of Health Sciences<br>Breckman, Trilby

Keywords:	Phobia, Children and adolescents, randomised controlled trial, one-session treatment, exposure therapy

SCHOLARONE™
Manuscripts

ALLEVIATING SPECIFIC PHOBIAS EXPERIENCED BY CHILDREN TRIAL (ASPECT)

Clinical and Cost-effectiveness of One Session Treatment (OST) vs. Multi-session Cognitive Behaviour Therapy (CBT) for Specific Phobias in Children: Protocol for a Non-inferiority Randomised Controlled Trial

Barry Wright[1], Alexander J Scott[2], Cindy Cooper[2], David Marshall[4], Lucy Tindall[1], Thompson Davis[8], Dawn Teare[2], Ellen Lee[2], Karina Lovell[5], Penny Bee[5], Lina Gega[3], Rebecca Hargate[1*], Jonathan Wilson[7], Shehzad Ali[3], Katie Biggs[2], Dean McMillan[3], and Trilby Breckman[6]

Word count: 5940

* Corresponding author Rebecca.hargate@nhs.net

[1]Child Oriented Mental Health Intervention Centre, IT Centre, Innovation Way, Heslington, York, YO10 5NP, UK

[2]ScHARR, The University of Sheffield, Regent Court, 30 Regent Street, Sheffield, S1 4DA, UK

[3] Department of Health Sciences & Hull York Medical School, University of York, Heslington, York, YO10 5DD, UK

[4]Centre for Reviews and Dissemination, University of York, Heslington, York, YO10 5DD, UK

[5] Division of Nursing, Midwifery and Social Work, University of Manchester, Oxford Road, Manchester. M13 9PL, UK

[6]Triumph Over Phobia (TOP UK), PO Box 3760, Bath, BA2 3WY, UK

[7] Norfolk and Suffolk NHS Foundation Trust, 80 St Stephens Rd, Norwich, NR1 3RE, UK

[8]Louisiana State University, Baton Rouge, LA, 70803, USA

For peer review only - http://bmjopen.bmj.com/site/about/guidelines.xhtml

ALLEVIATING SPECIFIC PHOBIAS EXPERIENCED BY CHILDREN TRIAL (ASPECT)

Abstract

Introduction: Specific phobias (intense, enduring fears of an object or situation that lead to avoidance and severe distress) are highly prevalent among children and young people. Cognitive Behaviour Therapy (CBT) is a well-established, effective intervention, but it can be time-consuming and costly because it is routinely delivered over multiple sessions during several months. Alternative methods of treating severe and debilitating phobias in children are needed, like One-Session Treatment (OST), not only to reduce time and cost, but also to prevent therapeutic drift and help children recover quickly. Our study explores whether (a) outcomes with OST are 'no worse' than outcomes with multi-session CBT, (b) OST is acceptable to children, their parents and the practitioners who use it, and (c) OST offers good value for money to the National Health Service (NHS) and to society. **Method**: A pragmatic, non-inferiority, randomised controlled trial (RCT) will compare OST with multi-session CBT based therapy on their clinical and cost-effectiveness. The primary clinical outcome is a standardised behavioural task of approaching the feared stimulus at six-months post randomisation. The outcomes for the within-trial cost-effectiveness analysis are quality-adjusted life years (QALYs) based on EQ-5D-Y, and individual-level costs based of the intervention and use of health and social service care. A nested qualitative evaluation will explore children's, parents' and practitioners' perceptions and experiences of OST. A total of 286 children, 7-16 years old, with DSM-IV diagnoses of specific phobia will be recruited via gatekeepers in the NHS, schools and voluntary youth services, and via public adverts. **Ethics and dissemination**: The trial received ethical approval from North East and York Research Ethics Committee (Reference: 17/NE/0012). Dissemination plans include publications in peer-reviewed journals, presentations in relevant research conferences, local research symposia, and seminars for children and their families, and for professionals and service managers. **Trial Registration Number:** ISRCTN19883421.

For peer review only - http://bmjopen.bmj.com/site/about/guidelines.xhtml

Keywords: phobia; children and adolescents; randomised controlled trial; one-session treatment; exposure therapy.

For peer review only

For peer review only - http://bmjopen.bmj.com/site/about/guidelines.xhtml

Strengths & Limitations

- To our knowledge, this is the largest proposed treatment study and economic evaluation of interventions for childhood phobias to-date.
- Interventions are delivered by multi-disciplinary professionals working in multiple sectors including health, education, and voluntary organisations.
- Children with all types of specific phobias and different learning abilities are included.
- There are no mid-point outcomes; therefore, the study cannot tell whether children improve with one-session treatment quicker than with multi-session therapy or how the two interventions may work in different ways to change outcomes (mechanisms of action). However, the study includes longer-term outcomes which are useful in evaluating sustained effect, if any, of the intervention.

For peer review only

For peer review only - http://bmjopen.bmj.com/site/about/guidelines.xhtml

ALLEVIATING SPECIFIC PHOBIAS EXPERIENCED BY CHILDREN TRIAL (ASPECT)

Introduction

A specific phobia is an intense, enduring fear of an identifiable object or situation that leads to distress and avoidance[1]. It is estimated that between 5% and 10% of children have a specific phobia severe enough to impact on their everyday functioning[2], but fewer than 10% seek treatment, therefore the average duration of phobias is 20 years[3]. The psychological, developmental and medical impact of these phobias is significant, with higher rates of health service usage than most other anxiety disorders[4]. Phobias in children can result in considerable academic difficulties[5], personal distress[6] and interference in day-to-day activities[7]. Cognitive Behavioural Therapy (CBT) is the dominant therapeutic intervention for specific phobias[8 9] within health services in the UK. Robust evidence supports the efficacy of CBT for anxiety disorders in general [10-12], and for specific phobias in particular; however, CBT can be time-consuming, costly, and difficult to access because it is delivered over multiple sessions during several weeks or months by therapists that have received lengthy training [13-16].

Alternative methods of CBT delivery, like One-Session Treatment (OST)[17], can be used to reduce demands on therapist time and associated costs, prevent therapeutic drift, and ultimately help children recover quicker [18 19]. OST is a variant of CBT, delivered in a single three-hour session. A central feature is graded exposure where the subject comes into proximity with the phobic stimulus in a series of graduated steps (from the easiest to the most difficult). For example, graded exposure for a phobia of dogs might first involve looking at pictures of dogs, followed by videos of dogs, then by observing dogs in a local park at a distance, and finally by interacting with dogs first hand. OST has been shown to be clinically effective in children[20-22]. For example, Ollendick and colleagues[20] randomised 196 children (ages 7 to 16) with phobias to one of three groups: (i) One Session Treatment; (ii) an education support group; or (iii) a wait list control group. OST was superior to both

For peer review only - http://bmjopen.bmj.com/site/about/guidelines.xhtml

comparators as measured by improved phobia severity, percentage of children who were diagnosis free, and child- and parent-rated anxiety and treatment satisfaction post-intervention and at six months follow-up. However, to our knowledge, OST has not been compared to the current 'gold-standard' approach to specific phobias (i.e., CBT), and has not been trialled in the UK within the National Health Service (NHS).

The present research is a non-inferiority, parallel group randomised controlled trial comparing the clinical and cost-effectiveness of OST vs. CBT for specific phobias in children and young people ages 7 to 16 years old. The primary aim is to test whether OST is non-inferior (i.e., produces *similar* clinical benefits) to multi-session CBT in helping children with their specific phobia(s), and the wider impact their phobia(s) have on mental health and quality of life. A nested qualitative evaluation will explore whether OST is acceptable to children, their parents, and the practitioners who deliver OST. Finally, an economic evaluation will examine whether OST offers good value for money to the NHS and society compared to multi-session CBT.

Methods and Analysis

Participants

The present research will recruit children and young people aged 7-16 years old with one or more specific phobias, as defined by the DSM-IV[1]. The DSM-IV criteria are: i) marked and out-of-proportion fear to a specific object or situation, ii) exposure to the feared object or situation provokes immediate anxiety, iii) the phobic situation is avoided where possible, iv) the avoidance or distress associated with the phobia interferes with the person's routine or functioning (e.g. learning, sleep, social activities) and v) the phobic avoidance or distress is present for 6 months or more. We include all types of phobias where we can introduce or safely simulate the stimulus for the purpose of exposure therapy. We exclude children who have conditions that are often referred to as "phobias" but are not *specific*

For peer review only - http://bmjopen.bmj.com/site/about/guidelines.xhtml

phobias (e.g. social or separation anxiety referred to in some circumstances as "school phobia") or when children need a different intervention as a priority (e.g. for psychosis or self-harm).

Design

This is a non-inferiority randomised controlled trial that compares clinical outcomes of OST with those of multi session CBT at 6 months post-randomisation. Nested within this trial is an economic evaluation of the cost-effectiveness of OST against CBT for outcomes and costs over 6 months. Interviews evaluate perceptions and experiences of OST by children, parents and practitioners also at 6 month follow-up. An internal 9-month pilot determines feasibility of recruitment and retention, and monitors the occurrence of any adverse events.

Recruitment

Our recruitment strategies follow routine care pathways for children with specific phobias. These pathways differ across our participating sites, which include NHS Trusts, Local Authorities and voluntary agencies across England. We have mapped the care pathways in each geographical area and we promote the study within teams of professionals who routinely receive referrals of children with specific phobias, such as Child and Adolescent Mental Health Services (CAMHS), Children's Hospitals, youth services and schools. We also draw attention of the study to potential participants through public adverts, e.g. in local newspapers and posters in GP surgeries.

Randomisation

Following written consent and completion of the baseline measures, random allocation to treatment groups is carried out remotely through a secure web-based program designed by the Sheffield Clinical Trials Unit (CTRU). The randomisation allocation ratio is 1:1 to facilitate equal sample sizes across both the OST and CBT groups. Randomisation is

For peer review only - http://bmjopen.bmj.com/site/about/guidelines.xhtml

stratified according to two age groups (7-11 years old vs. 12-16 years old) and two phobia severity groups (mild-to-moderate vs. severe); we use random permuted blocks of variable size to ensure enough participants are allocated evenly to each arm of the trial within each stratum.

Blinding

Complete concealment of allocation, “blinding”, from both participants and researchers in studies of psychological interventions is often extremely difficult, and, at times, impossible[23-26]. Our study takes a number of steps to facilitate blind assessment of outcomes as a way of reducing bias. First, the research assistants collecting baseline and follow-up outcomes are independent of the practitioners delivering the interventions. Second, the research assistants are not informed of the participants’ group allocation and are not involved in the therapy sessions. Participants are explicitly reminded not to disclose to the research assistant their allocated group. In the event of these procedures being compromised (e.g. a research assistant learning the participant’s allocated group), we record this and arrange for a different outcome assessor in the future wherever possible. The trial statisticians and health economists are blind to treatment allocation whilst the trial is ongoing.

Interventions

One-Session Treatment (OST)

OST is a variant of CBT, but rather than being delivered in weekly 45-60 min sessions as in conventional CBT, OST is delivered in a longer single session (approximately 3 hours). Prior to the single session, a 1-hour assessment/planning session takes place during which the practitioner carries out an assessment in order to plan the treatment session. This involves:

a) determining the nature, history and associations of the phobia.

For peer review only

b) determining any maintaining factors for the phobia (e.g. what the child avoids, what safety behaviours s/he engages in, how friends and family may collude with the phobia);

c) collecting information about the child's cognitions including catastrophic thoughts (e.g. what do you think may happen if…, what goes through your mind when you…);

d) generating a fear hierarchy (i.e. a 'ladder' of situations or objects that the child avoids because of their phobia, in the order of the fear or anxiety evoked, starting from the least frightening).

e) explaining what is involved in treatment and that the exposure tasks are graded and negotiated in every step of the way and that nothing will ever happen without the child's permission.

The single session combines graded exposure, participant modelling, reinforcement, and cognitive challenges. The main principle of graded exposure is that the child gradually confronts the situations from their fear hierarchy and remains in each situation until their anxiety/fear subsides at least by 50%. In participant modelling, the therapist first demonstrates to the child how to interact with the phobic object and then helps the child gradually approach the phobic object by joint approaches with the therapist gradually fading involvement. Reinforcement from the therapist may take the form of social praise, encouragement etc. Finally, the therapist uses the exposure tasks as means for actively eliciting, challenging, and testing negative cognitions or catastrophic thoughts associated with the feared situation and by promoting positive cognitions in phobic situations. It is important that the child maximises, maintains and generalises their gains from graded exposure by practising self-directed and parent/carer supported exposure tasks at home for the ensuing weeks. Maintenance tasks include a commitment to refrain from avoiding or escaping from feared situations and to deliberately engineer or enter a feared situation regularly.

For peer review only - http://bmjopen.bmj.com/site/about/guidelines.xhtml

Cognitive Behavioural Therapy (CBT)

CBT helps people to change unhelpful beliefs and behaviours arising in feared situations, such as catastrophic thinking and safety behaviours. Interventions based on CBT are the dominant model of treatment for specific phobias, and are supported by a robust literature attesting to its efficacy [8-12]. CBT interventions are often delivered in 45-60 minute sessions every week or fortnight over several weeks or months. Each CBT session takes the form of structured discussions with a therapist, has a specific agenda, and sets homework tasks for the child or young person between sessions. CBT for children and young people with specific phobias aims to help the child/young person to: (i) recognise anxious feelings and bodily reactions to anxiety; (ii) gradually confront their feared situations until their anxiety subsides; (iii) capture and challenge anxious thoughts when faced with a phobic situation or object; (iv) challenge avoidance behaviours, or behaviours that maintain or reinforce the phobia; and (v) develop coping strategies and anxiety management techniques if distress and physical responses to anxiety are overwhelming. There is no recommended number of CBT sessions for specific phobias but it can vary between 4 and 20 sessions.

Fidelity to the interventions

Practitioners responsible for specific phobia treatment across the recruiting centres will be trained in OST during a day's workshop, with senior practitioners (e.g. trained therapists) attending a modified 1/2 –day training on OST and a ½-day training on supervising and training others in OST to be able to offer continuous support and supervision to junior colleagues. OST and CBT sessions are audio-recorded whenever possible with consent from the child and parent. A random selection of 15% of participants at each site (or the nearest whole number) has one of their sessions (whether OST or CBT) reviewed by a supervisor at quarterly intervals. Supervisors score the recorded sessions to assess therapist fidelity to OST and CBT using the OST Rating Scale (OST-RS)[27] and the Cognitive

For peer review only - http://bmjopen.bmj.com/site/about/guidelines.xhtml

Behaviour Therapy Scale for Children and Young People CBTS-CYP, [28] respectively. This is to make sure that CBT principles are applied in both treatment arms. These are used in supervision to improve fidelity to the intervention and address training needs for the therapists as per usual practice.

Outcome Measures

We are collecting demographic information (e.g. age, gender etc.) from all children/young people and their parents. Children and parents are also asked if they have a treatment preference at baseline, although this does not affect their randomisation in any way. Participants complete a series of questionnaires at baseline and then at 6 months follow up, as described below.

Behavioural Avoidance Task (BAT)

The primary outcome is the BAT at six-months post randomisation. The BAT is a widely used measure of treatment outcomes for phobias in children[6 21]. The BATs for the ASPECT trial are standardised for each stimulus and have been found to have good test-retest reliability[20 29] and strong correlation with other outcome measures of anxiety and phobia[30 31]. During a BAT, the child gradually confronts their phobic stimulus over ten pre-defined steps of increasing difficulty; the child does not have to complete all the steps. For example, a child with spider phobia may start the BAT at step 0, standing outside of a room that has a spider contained in a box. Subsequent steps the child may take are: opening the door (step 1), passing through the doorway (step 2), through to holding the spider for 20 seconds (step 10). The number of steps the participant takes is the main unit of measurement recorded for analysis. The BAT also includes a measure of subjective units of distress (SUDs) whereby the child reports their level of fear at the start of the BAT, and at the last step completed (ranging from 0 = no fear at all to 8 = extreme fear).

For peer review only - http://bmjopen.bmj.com/site/about/guidelines.xhtml

The Anxiety Disorder Interview Schedule for Children and Parents (ADIS-C/P)

ADIS; [32] is a well-established semi-structured interview,[3 32 33] that obtains information from both the child and their parent/carer about the type of specific phobias experienced (e.g. animal/insect, small spaces, blood/injection phobias etc.), the degree of associated fear (rated from 0=not at all to 8=very, very much),whether the phobia causes avoidance (rated as 'yes' or 'no'), and interference with daily life (rated from 0=not at all to 8=very, very much).

Child Anxiety Impact Scale for Children and Parents (CAIS-C/P)

The CAIS-C/P; [34] collects information as to what extent feeling 'nervous, anxious or afraid' impacts on the daily life of the child. The child and the parent independently rate, on a 4-point scale ranging from 0=not at all to 4=very much, 27 statements grouped in 3 sub-domains: school activities (e.g. *getting to school on time in the morning*), social life (e.g. *making new friends*) and home/family life (e.g. *getting ready for bed at night*).

The Revised Children's Anxiety and Depression Scale (RCADS-C/P)

The RCADS-C/P[35-38] comprises 47 items to capture separation anxiety disorder (SAD), social phobia (SP), generalized anxiety disorder (GAD), panic disorder (PD), obsessive compulsive disorder (OCD), and major depressive disorder (MDD). Children state the frequency of which statements such as "*I worry about things*", *I have trouble sleeping*" and "*I feel I will make a fool of myself in front of people*" relate to them on a 4-point scale: 0=never, through to 1=sometimes, 2=often, and 3=always.

EQ-5D-Y

The EQ-5D-Y, originally developed by the EuroQoL group[39 40 41], is a widely used measure of health-related quality of life in children and young people. Children and young people are able to classify their own health on a three-point scale, 1=no problems, 2=some problems and 3=a lot of problems, over five dimensions: mobility (walking about), looking after myself, doing unusual activities, having pain or discomfort, and feeling worried, sad or

For peer review only - http://bmjopen.bmj.com/site/about/guidelines.xhtml

unhappy. Additionally, the EQ-5D-Y includes a visual analogue scale where participants can indicate their overall health status from 0 (worst imaginable state) to 100 (best imaginable state). All questions refer to the participant's health state "today".

Child Health Utility 9D (CHU-9D)

The CHU-9D is a health related quality of life measure validated for children and young people[42 43]. Participants select a sentence from a possible five to describe how they feel today in relation to a number of constructs (i.e. sadness, tiredness, pain, etc.). For example, participants are asked to select one of five statements that best reflects how worried they feel ranging from "*I don't feel worried today*" to "*I feel very worried today*".

Goal-based Outcome Measure

A goal-based outcome measure is a method of comparing how far a participant feels they have moved towards reaching a specific goal they have set before the intervention has begun. A goal-based outcome measure based on recent guidelines[44] will be used to set up to three goals at baseline, with progress towards meeting these goals rated on a 10-point scale at the 6-month follow-up point. Progress can range from 0 (i.e. goal not met), to 5 (i.e. half-way to reaching the goal), through to 10 (i.e. goal reached). For example, a participant with a dog phobia may set as their goal that they would like to be able to spend teatime at their grandmother's house where there is a dog.

Resource Utilisation Questionnaire

We developed a bespoke questionnaire to assess use of healthcare and other resources, based on a previous resource utilisation questionnaire by Barrett and colleagues[46] and its adaptations for younger populations by members of our research team [47 45 46]. The questionnaire is completed by the parent and collects data on resource and service utilisation relating to the child over the previous 6 months on: general health and community service use (i.e. appointments with GP's, nurses, social services and educational services); mental health

For peer review only - http://bmjopen.bmj.com/site/about/guidelines.xhtml

service use (i.e. appointments with a psychiatrist, psychotherapists, psychologists, CAMHS therapists and other forms of mental health support); hospital-based services (i.e. visits to A & E, urgent care centres and hospital stays); days missed from school by the child; days missed by parents/carers from work or studies.

Statistical Analysis

Sample Size

The proposed sample size and non-inferiority margin are based on two separate Cochrane reviews looking at the effects of psychotherapy for those experiencing anxiety. First, Wolitzky-Taylor and colleagues[47] conducted a review on studies that used both observer-rated BAT-like assessments and self-report questionnaires on adults with specific phobias and reported an overall, large effect size of $d = 0.81$. However, as the treatment may have a different effect on children, we also examined the review of Reynolds and colleagues[48]. This review was conducted on studies of children with specific phobias but used self-report questionnaires rather than observer-rated BATs. The review reported a large effect size ($d = 0.85$) for multi-session CBT. Prior meta-analyses suggest that a standardised mean difference of around 0.8 on the BAT scale is clinically important. Therefore, we set the non-inferiority margin to be half of this at 0.4[61]. Assuming a correlation of 0.5 between baseline and final BAT measure, we would require 200 participants (100 in each arm) to have 90% power with a 2.5% one-sided significance level to demonstrate non-inferiority of OST compared to CBT. The therapy is delivered by therapists who will see approximately 15 patients each and we anticipate a weak therapist effect (intraclass correlation coefficient (ICC = 0.01)). This clustering will lead to a design effect of 1.14 which increases the number required per arm to 114. We further assume a 20% dropout rate suggesting that total number of 286 children and young people (143 per arm) will need to be recruited to the study to demonstrate non-inferiority of OST compared to CBT.

For peer review only - http://bmjopen.bmj.com/site/about/guidelines.xhtml

Data Analysis

As this trial follows a randomised, parallel group, non-inferiority design, data will be analysed and reported according to both CONSORT guidelines[49] and the non-inferiority trials CONSORT extension[61]. Baseline data, including demographic characteristics (e.g. age, gender) and clinical measures (e.g. BAT, RCADS), will be reported between the two randomised groups. Jones and colleagues[50] recommend both per protocol and intention to treat (ITT) analyses for non-inferiority designs. For an equivalence or non-inferiority trial, as opposed to a comparative trial, ITT analyses are not considered conservative, as any blurring of the difference between the treatment groups increases the chance of declaring equivalence. We follow this recommendation with the refinement that the main analysis of the primary outcome will be per protocol (or completers only) with a sensitivity analysis on the ITT population[51]. We will require both the per protocol and ITT analyses to demonstrate conclusive evidence to declare that OST is "non-inferior" to multi-session CBT. If the results of the analysis are discrepant (e.g. the ITT rejects the null of inferiority but the per protocol analysis does not, or vice versa) then we will report the conflicting results from both analyses highlighting the inconclusive nature of the results. For the sake of the analysis a participant in the OST group is defined as per protocol if they attend both the initial functional assessment and the rapid exposure therapy session. A participant in the CBT group is defined as per protocol if they attend at least 4 CBT sessions.

The primary outcome (mean BAT score at 6 months) and the secondary outcomes (ADIS, CAIS, EQ-5D-Y, CHU-9D, RCADS and goal-based outcome scores) will be compared between groups using mixed effects linear regression with exchangeable correlation to allow for the clustering of outcomes within therapist. The analysis will be conducted controlling for baseline BAT score, site, and stratifying variables (age group and baseline phobia severity). The null hypothesis of inferiority will be rejected if the two sided

For peer review only - http://bmjopen.bmj.com/site/about/guidelines.xhtml

95% confidence interval (CI) for the difference is wholly below 0.4 (the range of clinical non-inferiority).

We anticipate some dropout/attrition, so case missing data may be an issue. Case and item missing data will be examined and multiple imputation methods will be used to reduce bias due to any missing responses in both the per protocol and the ITT analyses. Where appropriate, modelling methods that generate robust standard errors in the presence of missing data will be considered.

Economic Evaluation

We are conducting an economic evaluation from the UK personal and social services perspective. This will take the form of a within-trial cost-utility analysis to determine the incremental cost per unit of quality-adjusted life years (QALYs) of OST compared with CBT in children with specific phobias. Costs will be calculated based on: i) resources required to deliver the intervention and ii) individual-level use of health and social services by children and absenteeism from school for children or from work because of childcare for parents over the study period. Resource use will be multiplied by unit costs to arrive at total cost in each arm. QALYs will be calculated by measuring health-related quality of life using the self-reported EQ-5D-Y over the study period.

The resources required to deliver the OST intervention will be calculated using bottom-up estimation of the time spent by professionals as well as other resources used (including phobic stimulus acquisition, i.e. animal hire). Individual-level service use data will be based on self-reported use of primary and secondary health care as well as social care using a standardised resource utilisation questionnaire. Unit costs of health and social service use will be obtained from the UK national database of reference costs Department of Health, [52] and the Unit Costs of Health and Social Care report produced by the Personal Social

For peer review only - http://bmjopen.bmj.com/site/about/guidelines.xhtml

Services Research Unit[53]. The cost of medication will be based on the most recent version of the British National Formulary (BNF)Royal Pharmaceutical Society of Great Britain, [54].

Individual-level responses on the EQ-5D-Y will be used to estimate health related quality of life based on a UK population valuation set. Subsequently, an area under the curve approach will be used to calculate QALYs for each child. QALY is a utility-based measure, i.e. it measures each person's health state in terms of quality of life dimensions and then weights it on the value or utility of the health state based on UK population preferences[55].

The primary economic analysis will be cost-utility analysis conducted over the trial follow-up period (6 months). Total costs, including the intervention cost and service utilisation costs, and QALYs will be compared between the two interventions. Unadjusted costs and outcomes will be presented in a descriptive analysis using parametric and non-parametric tests. The statistical analysis will compare mean costs and QALYs using a regression model. The regression analysis will control for baseline differences in utility[56]. The specification of the model will follow the approach recommended by Glick, Doshi, Sonnad, and Polsky[57] which considers the distribution of the dependent variable as well as any correlation between the cost and QALY outcomes. The regression coefficient on treatment will then represent the difference in mean cost and mean QALYs between groups. A bootstrap method will be used to produce confidence intervals around the cost and QALY differences due to the likely skewness in the distribution of regression residuals[58]. To present this in the UK decision-making context, the results will be in the conventional form of a cost-effectiveness acceptability curve (CEAC). CEAC presents the probability of the intervention being cost-effective over a range of willingness-to-pay (WTP) thresholds per QALY[59]. The higher the probability, the more likely it is that the treatment is cost-effective at the particular WTP threshold.

For peer review only - http://bmjopen.bmj.com/site/about/guidelines.xhtml

The following sensitivity analyses will be conducted; i) a cost-utility analysis using the CHU9D instrument instead of EQ-5D; ii) a cost-effectiveness analysis using a phobia-specific measure instead of a utility-based measure; and iii) cost-utility analysis from a health services perspective.

Nested Qualitative Evaluation

We will invite a sub-sample of trial participants to participate in individual semi-structured interviews and use maximum variation sampling to ensure a spread of participants differing in age, gender, socio-economic background and type of phobia. We will obtain written consent (and assent where appropriate) to interview a sample of i) children receiving OST, ii) their parents/guardians and iii) practitioners delivering OST across all study sites. With parental consent. We will recruit and interview parents and children separately. Joint parent-child interviews will be included where participants prefer. The final sample size for the interviews in each participant group will be determined by data saturation, i.e. the point where no new themes, ideas and/or concepts emerge. Based on a previous nested qualitative evaluation of patient acceptability of brief psychological interventions[60], we estimate that we will need to complete a maximum of 30 interviews with parents, 25 with children and 15 with practitioners.

Interviews with children and their parents will be conducted after participants have completed the final outcome measures at the 6-month follow-up point. Interviews for parents will focus on phobia experiences, personal and family impact, perceived treatment need, treatment expectations and treatment acceptability (e.g. content, delivery mode, format, setting and facilitation). Child interviews will focus on the same topics, adapted for age and developmental maturity. Face-to-face interviews with children will draw on the principles of 'draw and write' techniques, whereby children will be offered an opportunity to draw a picture relating to their experiences as a prompt to initiate more in-depth discussion[61 62].

For peer review only - http://bmjopen.bmj.com/site/about/guidelines.xhtml

Child interviews will last a maximum of 30 minutes and parent interviews a maximum of 60 minutes, with total time guided by the interviewees. Clinician interviews will last for a maximum of 60 minutes and focus on their experiences and views of delivering OST, the individual and organisational support required and the perceived suitability OST for their patient group.

Interviews with parents and older children (13 years plus) will be conducted face-to-face in treatment settings or at participants' homes, or via the telephone, depending upon participant preference. Interviews with younger children (12 years and under) will be conducted face-to-face in treatment settings or at participants' homes, depending upon participant preference. Practitioner interviews will take place face-to-face in their workplace or over the telephone once their own involvement in the trial is complete.

All interviews will be digitally recorded and transcribed verbatim. Analysis will follow a qualitative framework approach[63], a widely used method of analysing primary qualitative data pertaining to health care practices with policy relevance[64]. Framework analysis permits both deductive and inductive coding, enabling potentially important themes or concepts that have been identified a priori to be combined with additional themes emerging de novo.

Data coding will be undertaken independently by two trained researchers. We will additionally train a Patient and Public Involvement (PPI) representative to code alongside these researchers to ensure coding takes account of potential differences in perspective. Coders will meet regularly to develop a shared coding manual and to ensure that all emerging codes remain grounded in original data. An Excel spread sheet will be developed which will incorporate preliminary framework themes as column headings and the demographic information related to participants who provided data under each theme. As the constant comparison of new data occurs and the coding team's understandings of the themes under

For peer review only - http://bmjopen.bmj.com/site/about/guidelines.xhtml

consideration develop, the framework will be amended and re-shaped to enable the introduction of new codes and/or the deletion of redundant, similar or otherwise compromised codes. In this way, a final framework will be achieved that is considered representative of the entire dataset. We will code data from each stakeholder group (children, parents/guardians and practitioners) separately before comparing and contrasting data findings across groups. The final coding manuals for each participant group will be presented with example entries, to the project's advisory committees to confirm validity, coherence and conceptual relevance.

Public and Patient Involvement

The research protocol was developed in partnership with service user groups and was reviewed in terms of both feasibility and relevance. In addition to this, the views of relevant charitable organisations were sought and a representative from the Triumph Over Phobia UK support group is a co-applicant on this application.

We have consulted with PPI representatives about their views on the full research proposal and how best to involve patients throughout the proposed project. In particular, we have discussed comments after the first round application with representatives of Triumph Over phobia. This includes issues around the non-inferiority design, how to measure fidelity and contamination.

We have also engaged the York Youth Council, who have commented on this application and discussed the methodology of the trial and the potential impact of the therapy on the community.

We see PPI as central to our research. A representative from Triumph Over Phobia are on our trial management group and actively involved in the planning and delivery of this research. Applicants KL and PB are named applicants (PI-and Co-PI respectively) on an NIHR-funded programme grant seeking to improve user involvement in mental health care planning. This

For peer review only - http://bmjopen.bmj.com/site/about/guidelines.xhtml

programme has been recognised by the MHRN for exemplary carer involvement. Applicant PB has substantial experience of training users and carers in research methodologies, interviewing skills and qualitative data analysis. We recognise the need for independent qualitative data analysis, and will train a service user researcher. The service user representative will be reimbursed for their time, commensurate with current INVOLVE guidelines.

Ethics and dissemination

Obtaining informed consent from participants

Written informed consent from children and parents is taken at a face-to-face meeting with a research assistant. As all participants in this study are aged 16 or under, consent is required from both a person with parental responsibility, and the participant themselves if they are deemed competent to do so. Where a child is not deemed competent to give full informed consent, we take parental consent and seek assent from the child. Prior to the consent meeting, the parents and the children receive information about the study and have the opportunity to discuss any questions or concerns with members of the research team. Two age-specific versions of the child information sheets are available: one for those aged 7 to 11 years and one for those aged 12 to 16 years. All information leaflets and consent forms are co-developed by the research team and PPI representatives to ensure acceptability amongst participants. If a participant wishes to withdraw from their allocated intervention, we check whether they are still agreeable to participate in the planned follow-up assessment at 6 months, so that their outcomes can be included within our ITT analysis.

Data management

Trial data are extracted from source documents and entered onto the CTRU's in-house data management system (Prospect). Prospect stores data in a PostgreSQL database hosted by the University of Sheffield. The database uses industry standard techniques to provide

For peer review only - http://bmjopen.bmj.com/site/about/guidelines.xhtml

security, including password authentication and encryption using SSL/TLS. Access to Prospect is controlled by usernames and encrypted passwords. A comprehensive privilege management feature ensures only the minimum amount of data required is available to each individual to complete their tasks. The system has a full electronic audit trail and is regularly backed up. Access to data is restricted to users with appropriate privileges only. All data are collected and retained in accordance with the Data Protection Act 1998 and Sheffield Clinical Trial Research Unit (CTRU) standard operating procedures (SOPs).

Research governance and conduct of the trial

Trial monitoring procedures and site monitoring are undertaken at a level appropriate to a risk assessment performed by the Trial Sponsor (Leeds and York Partnership NHS Foundation Trust, 2150 Century Way, Thorpe Park, Leeds, LS15 8ZB) and the Sheffield Clinical Trial Research Unit (CTRU). Three committees govern the conduct of this study, in accordance with Sheffield CTRU's Standard Operating Procedures (SOPs): a Trial Steering Committee (TSC), an independent Data Monitoring and Ethics Committee (DMEC) and a Trial Management Group (TMG)

The TSC consists of an independent Chair, an independent subject specialist, an independent clinical academic, an independent statistician and a patient representative. The TSC meets approximately every 6 months from the start of the trial. The DMEC consists of an independent chair, an independent statistician, and an independent ethics specialist experienced in research with children and families. The TMG comprises the co-applicants and the two trial managers who are jointly supervised by the CI, the Director of the Sheffield CTRU and a lead trial manager in the CTRU. Meeting attendance of the co-applicants depends on each meeting's agenda and relevance to their role.

For peer review only - http://bmjopen.bmj.com/site/about/guidelines.xhtml

Adverse events

An adverse events monitoring form is used to record any untoward occurrence affecting the participant after each therapy session by the therapist and at follow-up by the research assistant. Such an event can be directly related, possibly related or unrelated to the intervention. An occurrence is recorded if it is suspected to be related to the intervention or an aspect of the research procedures; the therapist assesses relatedness and research assistants seek advice from the site Principal Investigator. The occurrence of adverse events during the trial is monitored by the trial oversight and management groups, particularly the DMEC and the TSC. All adverse events are assessed for seriousness, and will be recorded as a Serious Adverse Event (SAE) if they are life-threatening, or require hospitalisation or prolongation of existing inpatient stay, or result in persistent or significant disability or incapacity or death.

Ethical approval

This study has received ethical approval from the North East - York Research Ethics Committee (Reference: 17/NE/0012). No pharmaceutical compounds or medical devices are used in this trial, therefore Clinical Trials Authorisation is not required.

Dissemination of the research findings

We will publish the study's findings in peer reviewed academic journals and present at local, national, and international conferences where possible. Furthermore, we will publish a short summary of the results on the ASPECT study website that can be accessed by all trial participants as well as relevant interest groups, including patient groups. Finally, towards the end of the trial, our Patient and Public Involvement (PPI) representatives will organize a meeting with stakeholders including parents and professionals working with young people with anxiety disorders to specifically discuss the dissemination of the study findings and put together a dissemination plan.

For peer review only - http://bmjopen.bmj.com/site/about/guidelines.xhtml

Trial status

Protocol V3 24/04/17. Recruitment to the trial began in June 2017 and is estimated to be completed in July 2019, with the final follow up assessments completed in March 2020.

Author Contributions

BW, DM, TD, LT, LG,DM, KL,PB, RH & CC designed the study and drafted the original grant proposal BW & CC provided trial leadership. CC, KB & RH provide governance oversight. CC, BW, DM, DMc, DT, KL, TD provide methodological expertise. SA provides health economics expertise. DT & EL provide statistical and data management expertise. LG, DMc, KL, JW and BW provide clinical expertise. TD, BW and LG provide training and supervision in OST and CBT. PB provides qualitative expertise. AS & LT are the trial's managers. TB leads on public and service user representation. All authors contributed to the writing and review of the trial's protocol. All co-authors approved the final version of this manuscript.

Funding

This article presents independent research funded by the National Institute for Health Research (NIHR) under its Health Technology Assessment (HTA) Programme (Grant Reference Number HTA15/38/04). The views expressed are those of the author(s) and not necessarily those of the NHS, the NIHR or the Department of Health.

Competing Interests

Dr Thompson Davis receives royalties from the sale of a book on OST. There are no competing interests for the other authors.

For peer review only - http://bmjopen.bmj.com/site/about/guidelines.xhtml

References

1. Association AP, Association AP. DSM-IV-TR: Diagnostic and statistical manual of mental disorders, text revision. *Washington, DC: American Psychiatric Association* 2000;75:78-85.

2. Kessler R, Berglund P, Demler O, et al. Lifetime prevalence and age-of-onset distributions of DSM-IV disorders in the national comorbidity survey replication. *Archives of General Psychiatry* 2005;62:593-602.

3. Stinson FS, Dawson DA, Chou SP, et al. The epidemiology of DSM-IV specific phobia in the USA: results from the National Epidemiologic Survey on Alcohol and Related Conditions. *Psychological Medicine* 2007;37(7):1047-59. doi: 10.1017/s0033291707000086

4. Deacon B, Lickel J, Abramowitz JS. Medical utilization across the anxiety disorders. *Journal of Anxiety Disorders* 2008;22(2):344-50. doi: 10.1016/j.janxdis.2007.03.004

5. Ialongo N, Edelsohn G, Werthamerlarsson L, et al. The significance of self-reported anxious symptoms in first-grade children - prediction to anxious symptoms and adaptive functioning in 5th grade. *Journal of Child Psychology and Psychiatry and Allied Disciplines* 1995;36(3):427-37. doi: 10.1111/j.1469-7610.1995.tb01300.x

6. Ollendick TH, March JS. *Phobic and anxiety disorders in children and adolescents: A clinician's guide to effective psychosocial and pharmacological interventions*. USA: Oxford University Press 2004.

7. Ollendick TH, King NJ, Muris P. Phobias in children and adolescents. In: Maj M, Akiskal HS, Lopez-Ibor JJ, et al., eds. Phobias New York: Wiley 2004:245-79.

8. Kendall PC, Hedtke KA. Cognitive-Behavioural Therapy for anxious children: Therapist manual. Ardmore: Workbook Publishing 2006.

For peer review only - http://bmjopen.bmj.com/site/about/guidelines.xhtml

9. Kendall PC, Robin JA, Hedtke KA, et al. Considering CBT with anxious youth? Think exposures. *Cognitive and Behavioral Practice* 2005;12(1):136-48. doi: 10.1016/s1077-7229(05)80048-3

10. Hofmann SG, Smits JAJ. Cognitive-behavioral therapy for adult anxiety disorders: A meta-analysis of randomized placebo-controlled trials. *Journal of Clinical Psychiatry* 2008;69(4):621-32.

11. Butler AC, Chapman JE, Forman EM, et al. The empirical status of cognitive-behavioral therapy: A review of meta-analyses. *Clinical Psychology Review* 2006;26(1):17-31. doi: 10.1016/j.cpr.2005.07.003

12. Hofmann SG, Asnaani A, Vonk IJJ, et al. The Efficacy of Cognitive Behavioral Therapy: A Review of Meta-analyses. *Cognitive Therapy and Research* 2012;36(5):427-40. doi: 10.1007/s10608-012-9476-1

13. Aschim B, Lundevall S, Martinsen EW, et al. General practitioners' experiences using cognitive behavioural therapy in general practice: A qualitative study. *Scandinavian Journal of Primary Health Care* 2011;29(3):176-80. doi: 10.3109/02813432.2011.595582

14. Wiebe E, Greiver M. Using cognitive behavioural therapy in practice: qualitative study of family physicians' experiences. *Canadian family physician Medecin de famille canadien* 2005;51:992-3.

15. Cavanagh K. Geographic Inequity in the Availability of Cognitive Behavioural Therapy in England and Wales: A 10-Year Update. *Behavioural and Cognitive Psychotherapy* 2014;42(4):497-501. doi: 10.1017/s1352465813000568

16. Shapiro DA, Cavanagh K, Lomas H. Geographic inequity in the availability of cognitive behavioural therapy in england and wales. *Behavioural and Cognitive Psychotherapy* 2003;31(2):185-92. doi: 10.1017/s1352465803002066

For peer review only - http://bmjopen.bmj.com/site/about/guidelines.xhtml

17. Öst LG. One-session treatment for specific phobias. *Behaviour Research and Therapy* 1989;27(1):1-7. doi: 10.1016/0005-7967(89)90113-7

18. Bower P, Gilbody S. Stepped care in psychological therapies: access, effectiveness and efficiency - Narrative literature review. *British Journal of Psychiatry* 2005;186:11-17. doi: 10.1192/bjp.186.1.11

19. National Collaborating Centre for Mental Health. Generalised anxiety disorder in adults: Management in primary, secondary and community care: British Psychological Society 2011.

20. Ollendick TH, Öst LG, Reuterskiöld L, et al. One-Session Treatment of Specific Phobias in Youth: A Randomized Clinical Trial in the United States and Sweden. *Journal of Consulting and Clinical Psychology* 2009;77(3):504-16. doi: 10.1037/a0015158

21. Öst LG, Svensson L, Hellstrom K, et al. One-session treatment of specific phobias in youths: A randomized clinical trial. *Journal of Consulting and Clinical Psychology* 2001;69(5):814-24. doi: 10.1037/0022-006x.69.5.814

22. Ryan SM, Strege MV, Oar EL, et al. One session treatment for specific phobias in children: Comorbid anxiety disorders and treatment outcome. *Journal of Behavior Therapy and Experimental Psychiatry* 2016;54:128-34.

23. Boutron I, Estellat C, Ravaud P. A review of blinding in randomized controlled trials found results inconsistent and questionable. *Journal of Clinical Epidemiology* 2005;58(12):1220-26. doi: 10.1016/j.clinepi.2005.04.006

24. Boutron I, Guittet L, Estellat C, et al. Reporting methods of blinding in randomized trials assessing nonpharmacological treatments. *Plos Medicine* 2007;4(2):370-80. doi: 10.1371/journal.pmed.0040061

For peer review only - http://bmjopen.bmj.com/site/about/guidelines.xhtml

25. Fergusson D, Glass KC, Waring D, et al. Turning a blind eye: the success of blinding reported in a random sample of randomised, placebo controlled trials. *British Medical Journal* 2004;328(7437):432-34. doi: 10.1136/bmj.37952.631667.EE

26. Sackett DL. Turning a blind eye - Why we don't test for blindness at the end of our trials. *British Medical Journal* 2004;328(7448):1136-36. doi: 10.1136/bmj.328.7448.1136-a

27. Ollendick TH, Halldorsdottir T, Fraire MG, et al. Specific phobias in youth: A randomized controlled trial comparing one-session treatment to a parent-augmented one-session treatment. *Behavior therapy* 2015;46(2):141-55.

28. Stallard P, Myles P, Branson A. The cognitive behaviour therapy scale for children and young people (CBTS-CYP): Development and psychometric properties. *Behavioural and cognitive psychotherapy* 2014;42(03):269-82.

29. Ollendick TH, Allen B, Benoit K, et al. The tripartite model of fear in children with specific phobias: Assessing concordance and discordance using the behavioral approach test. *Behaviour Research and Therapy* 2011;49(8):459-65. doi: 10.1016/j.brat.2011.04.003

30. Kindt M, Brosschot JF, Muris P. Spider phobia questionnaire for children (SPQ-C): A psychometric study and normative data. *Behaviour Research and Therapy* 1996;34(3):277-82. doi: 10.1016/0005-7967(95)00069-0

31. Boschen MJ, Veale D, Ellison N, et al. The emetophobia questionnaire (EmetQ-13): Psychometric validation of a measure of specific phobia of vomiting (emetophobia). *Journal of Anxiety Disorders* 2013;27(7):670-77. doi: 10.1016/j.janxdis.2013.08.004

32. Silverman WK, Albano AM. Anxiety Disorders Interview Schedule (ADIS-IV) Child and Parent Interview Schedules. Albany, NY: Greywind Publications 1996.

33. Silverman WK, Saavedra LM, Pina AA. Test-retest reliability of anxiety symptoms and diagnoses with the anxiety disorders interview schedule for DSM-IV: Child and

For peer review only - http://bmjopen.bmj.com/site/about/guidelines.xhtml

parent versions. *Journal of the American Academy of Child and Adolescent Psychiatry* 2001;40(8):937-44. doi: 10.1097/00004583-200108000-00016

34. Langley AK, Bergman RL, McCracken J, et al. Impairment in childhood anxiety disorders: Preliminary examination of the child anxiety impact scale-parent version. *Journal of Child and Adolescent Psychopharmacology* 2004;14(1):105-14. doi: 10.1089/104454604773840544

35. Chorpita BF, Yim L, Moffitt C, et al. Assessment of symptoms of DSM-IV anxiety and depression in children: A revised child anxiety and depression scale. *Behaviour research and therapy* 2000;38(8):835-55.

36. Chorpita BF, Moffitt CE, Gray J. Psychometric properties of the Revised Child Anxiety and Depression Scale in a clinical sample. *Behaviour research and therapy* 2005;43(3):309-22.

37. de Ross RL, Gullone E, Chorpita BF. The revised child anxiety and depression scale: a psychometric investigation with Australian youth. *Behaviour Change* 2002;19(02):90-101.

38. Bouvard M, Denis A. Reliability of the test-retest of the Revised Child Anxiety and Depression Scale (RCADS) and the assessment grid of the revised version of the Scale for Child Anxiety Related Emotional Disorders (SCARED-R). *L'Encéphale* 2012;38(6):524.

39. EuroQol Group. EuroQol-a new facility for the measurement of health-related quality of life. *Health policy (Amsterdam, Netherlands)* 1990;16(3):199-208.

40. Ravens-Sieberer U, Wille N, Badia X, et al. Feasibility, reliability, and validity of the EQ-5D-Y: results from a multinational study. *Quality of Life Research* 2010;19(6):887-97. doi: 10.1007/s11136-010-9649-x

For peer review only - http://bmjopen.bmj.com/site/about/guidelines.xhtml

41. Burstrom K, Bartonek A, Brostrom EW, et al. EQ-5D-Y as a health-related quality of life measure in children and adolescents with functional disability in Sweden: testing feasibility and validity. *Acta Paediatrica* 2014;103(4):426-35. doi: 10.1111/apa.12557

42. Ratcliffe J, Stevens K, Flynn T, et al. An assessment of the construct validity of the CHU9D in the Australian adolescent general population. *Quality of Life Research* 2012;21(4):717-25. doi: 10.1007/s11136-011-9971-y

43. Stevens K. Valuation of the Child Health Utility 9D Index. *Pharmacoeconomics* 2012;30(8):729-47.

44. Law D, Jacob J. Goals and Goal Based Outcomes (GBOs). 3rd ed. London: CAMHS Press 2013.

45. Wright B, Tindall L, Littlewood E, et al. Computerised cognitive behaviour therapy for depression in adolescents: study protocol for a feasibility randomised controlled trial. *Bmj Open* 2014;4(10) doi: 10.1136/bmjopen-2014-006488

46. Marshall D, Wright B, Allgar V, et al. Social Stories in mainstream schools for children with autism spectrum disorder: a feasibility randomised controlled trial. *BMJ open* 2016;6(8):e011748.

47. Wolitzky-Taylor KB, Horowitz JD, Powers MB, et al. Psychological approaches in the treatment of specific phobias: A meta-analysis. *Clinical Psychology Review* 2008;28(6):1021-37. doi: 10.1016/j.cpr.2008.02.007

48. Reynolds S, Wilson C, Austin J, et al. Effects of psychotherapy for anxiety in children and adolescents: A meta-analytic review. *Clinical Psychology Review* 2012;32(4):251-62. doi: 10.1016/j.cpr.2012.01.005

49. Schulz KF, Altman DG, Moher D. CONSORT 2010 statement: updated guidelines for reporting parallel group randomized trials. *Annals of internal medicine* 2010;152(11):726-32.

For peer review only - http://bmjopen.bmj.com/site/about/guidelines.xhtml

50. Jones B, Jarvis P, Lewis JA, et al. Trials to assess equivalence: The importance of rigorous methods. *British Medical Journal* 1996;313(7048):36-39.

51. Piaggio G, Elbourne DR, Pocock SJ, et al. Reporting of Noninferiority and Equivalence Randomized Trials Extension of the CONSORT 2010 Statement. *Jama-Journal of the American Medical Association* 2012;308(24):2594-604. doi: 10.1001/jama.2012.87802

52. Department of Health. Reference Costs Guidance 2015 to 2016. In: Health Do, ed., 2016:1 - 130.

53. Curtis L, Burns A. Unit Costs of Health and Social Care 2015. In: Unit PSSR, ed. Canterbury: University of Kent, 2015.

54. Royal Pharmaceutical Society of great Britain. British national formulary 72: Royal Pharmaceutical Society 2016.

55. Whitehead SJ, Ali S. Health outcomes in economic evaluation: the QALY and utilities. *British medical bulletin* 2010;96(1):5-21.

56. Manca A, Hawkins N, Sculpher MJ. Estimating mean QALYs in trial-based cost-effectiveness analysis: the importance of controlling for baseline utility. *Health Economics* 2005;14(5):487-96. doi: 10.1002/hec.944

57. Glick HA, Doshi JA, Sonnad SS, et al. Economic evaluation in clinical trials. Oxford: OUP 2014.

58. Hoch JS, Briggs AH, Willan AR. Something old, something new, something borrowed, something blue: a framework for the marriage of health econometrics and cost-effectiveness analysis. *Health Economics* 2002;11(5):415-30. doi: 10.1002/hec.678

59. Fenwick E, O'Brien BJ, Briggs A. Cost-effectiveness acceptability curves - facts, fallacies and frequently asked questions. *Health Economics* 2004;13(5):405-15. doi: 10.1002/hec.903

For peer review only - http://bmjopen.bmj.com/site/about/guidelines.xhtml

60. Lovell K, Bower p, Gellatly J, et al. Clinical effectiveness, cost-effectiveness and acceptability of low-intensity interventions in the management of obsessive-compulsive disorder: The obsessive-compulsive treatment efficacy randomised controlled trial (OCTET). *NIHR Journals Library* in press

61. Angell C, Alexander J, Hunt JA. 'Draw, write and tell': A literature review and methodological development on the 'draw and write' research method. *Journal of Early Childhood Research* 2015;13(1):17-28. doi: 10.1177/1476718x14538592

62. McWhirter J. The draw and write technique as a versatile tool for researching children's understanding of health and well-being. *International Journal of Health Promotion and Education* 2014;52(5):250-59.

63. Ritchie J, Spencer L, Bryman A, et al. Qualitative Data Analysis for Applied Policy Research. In: Bryman A, Burgess RG, eds. Analyzing Qualitative Data. London: Routledge 1994.

64. Dixon-Woods M. Using framework-based synthesis for conducting reviews of qualitative studies. *BMC medicine* 2011;9(1):1.

For peer review only

For peer review only - http://bmjopen.bmj.com/site/about/guidelines.xhtml

Reporting checklist for protocol of a clinical trial.

Based on the SPIRIT guidelines.

Instructions to authors

Complete this checklist by entering the page numbers from your manuscript where readers will find each of the items listed below.

Your article may not currently address all the items on the checklist. Please modify your text to include the missing information. If you are certain that an item does not apply, please write "n/a" and provide a short explanation.

Upload your completed checklist as an extra file when you submit to a journal.

In your methods section, say that you used the SPIRIT reporting guidelines, and cite them as:

Chan A-W, Tetzlaff JM, Altman DG, Laupacis A, Gøtzsche PC, Krleža-Jerić K, Hróbjartsson A, Mann H, Dickersin K, Berlin J, Doré C, Parulekar W, Summerskill W, Groves T, Schulz K, Sox H, Rockhold FW, Rennie D, Moher D. SPIRIT 2013 Statement: Defining standard protocol items for clinical trials. Ann Intern Med. 2013;158(3):200-207

		Reporting Item	Page Number
Title	#1	Descriptive title identifying the study design, population, interventions, and, if applicable, trial acronym	1
Trial registration	#2a	Trial identifier and registry name. If not yet registered, name of intended registry	2
Trial registration: data set	#2b	All items from the World Health Organization Trial Registration Data Set	2
Protocol version	#3	Date and version identifier	23
Funding	#4	Sources and types of financial, material, and other support	23
Roles and responsibilities: contributorship	#5a	Names, affiliations, and roles of protocol contributors	23
Roles and responsibilities:	#5b	Name and contact information for the trial sponsor	21

For peer review only

sponsor contact information			
Roles and responsibilities: sponsor and funder	#5c	Role of study sponsor and funders, if any, in study design; collection, management, analysis, and interpretation of data; writing of the report; and the decision to submit the report for publication, including whether they will have ultimate authority over any of these activities	21
Roles and responsibilities: committees	#5d	Composition, roles, and responsibilities of the coordinating centre, steering committee, endpoint adjudication committee, data management team, and other individuals or groups overseeing the trial, if applicable (see Item 21a for data monitoring committee)	21
Background and rationale	#6a	Description of research question and justification for undertaking the trial, including summary of relevant studies (published and unpublished) examining benefits and harms for each intervention	5
Background and rationale: choice of comparators	#6b	Explanation for choice of comparators	10
Objectives	#7	Specific objectives or hypotheses	6
Trial design	#8	Description of trial design including type of trial (eg, parallel group, crossover, factorial, single group), allocation ratio, and framework (eg, superiority, equivalence, non-inferiority, exploratory)	7
Study setting	#9	Description of study settings (eg, community clinic, academic hospital) and list of countries where data will be collected. Reference to where list of study sites can be obtained	7
Eligibility criteria	#10	Inclusion and exclusion criteria for participants. If applicable, eligibility criteria for study centres and individuals who will perform the interventions (eg, surgeons, psychotherapists)	6
Interventions: description	#11a	Interventions for each group with sufficient detail to allow replication, including how and when they will be administered	8

For peer review only

Interventions: modifications	#11b	Criteria for discontinuing or modifying allocated interventions for a given trial participant (eg, drug dose change in response to harms, participant request, or improving / worsening disease)	See note 1
Interventions: adherance	#11c	Strategies to improve adherence to intervention protocols, and any procedures for monitoring adherence (eg, drug tablet return; laboratory tests)	10
Interventions: concomitant care	#11d	Relevant concomitant care and interventions that are permitted or prohibited during the trial	N/A
Outcomes	#12	Primary, secondary, and other outcomes, including the specific measurement variable (eg, systolic blood pressure), analysis metric (eg, change from baseline, final value, time to event), method of aggregation (eg, median, proportion), and time point for each outcome. Explanation of the clinical relevance of chosen efficacy and harm outcomes is strongly recommended	9
Participant timeline	#13	Time schedule of enrolment, interventions (including any run-ins and washouts), assessments, and visits for participants. A schematic diagram is highly recommended (see Figure)	N/A
Sample size	#14	Estimated number of participants needed to achieve study objectives and how it was determined, including clinical and statistical assumptions supporting any sample size calculations	14
Recruitment	#15	Strategies for achieving adequate participant enrolment to reach target sample size	7
Allocation: sequence generation	#16a	Method of generating the allocation sequence (eg, computer-generated random numbers), and list of any factors for stratification. To reduce predictability of a random sequence, details of any planned restriction (eg, blocking) should be provided in a separate document that is unavailable to those who enrol participants or assign interventions	7
Allocation concealment	#16b	Mechanism of implementing the allocation sequence (eg, central telephone; sequentially numbered, opaque, sealed	8

For peer review only

mechanism		envelopes), describing any steps to conceal the sequence until interventions are assigned	
Allocation: implementation	#16c	Who will generate the allocation sequence, who will enrol participants, and who will assign participants to interventions	7
Blinding (masking)	#17a	Who will be blinded after assignment to interventions (eg, trial participants, care providers, outcome assessors, data analysts), and how	8
Blinding (masking): emergency unblinding	#17b	If blinded, circumstances under which unblinding is permissible, and procedure for revealing a participant's allocated intervention during the trial	8
Data collection plan	#18a	Plans for assessment and collection of outcome, baseline, and other trial data, including any related processes to promote data quality (eg, duplicate measurements, training of assessors) and a description of study instruments (eg, questionnaires, laboratory tests) along with their reliability and validity, if known. Reference to where data collection forms can be found, if not in the protocol	11
Data collection plan: retention	#18b	Plans to promote participant retention and complete follow-up, including list of any outcome data to be collected for participants who discontinue or deviate from intervention protocols	16
Data management	#19	Plans for data entry, coding, security, and storage, including any related processes to promote data quality (eg, double data entry; range checks for data values). Reference to where details of data management procedures can be found, if not in the protocol	21
Statistics: outcomes	#20a	Statistical methods for analysing primary and secondary outcomes. Reference to where other details of the statistical analysis plan can be found, if not in the protocol	15
Statistics: additional analyses	#20b	Methods for any additional analyses (eg, subgroup and adjusted analyses)	15
Statistics: analysis population and missing data	#20c	Definition of analysis population relating to protocol non-adherence (eg, as randomised analysis), and any statistical methods to handle missing data (eg, multiple imputation)	15

For peer review only

Data monitoring: formal committee	#21a	Composition of data monitoring committee (DMC); summary of its role and reporting structure; statement of whether it is independent from the sponsor and competing interests; and reference to where further details about its charter can be found, if not in the protocol. Alternatively, an explanation of why a DMC is not needed	21
Data monitoring: interim analysis	#21b	Description of any interim analyses and stopping guidelines, including who will have access to these interim results and make the final decision to terminate the trial	21
Harms	#22	Plans for collecting, assessing, reporting, and managing solicited and spontaneously reported adverse events and other unintended effects of trial interventions or trial conduct	22
Auditing	#23	Frequency and procedures for auditing trial conduct, if any, and whether the process will be independent from investigators and the sponsor	21
Research ethics approval	#24	Plans for seeking research ethics committee / institutional review board (REC / IRB) approval	22
Protocol amendments	#25	Plans for communicating important protocol modifications (eg, changes to eligibility criteria, outcomes, analyses) to relevant parties (eg, investigators, REC / IRBs, trial participants, trial registries, journals, regulators)	21
Consent or assent	#26a	Who will obtain informed consent or assent from potential trial participants or authorised surrogates, and how (see Item 32)	20
Consent or assent: ancillary studies	#26b	Additional consent provisions for collection and use of participant data and biological specimens in ancillary studies, if applicable	N/A
Confidentiality	#27	How personal information about potential and enrolled participants will be collected, shared, and maintained in order to protect confidentiality before, during, and after the trial	21
Declaration of interests	#28	Financial and other competing interests for principal investigators for the overall trial and each study site	23
Data access	#29	Statement of who will have access to the final trial dataset,	21

For peer review only

		and disclosure of contractual agreements that limit such access for investigators	
Ancillary and post trial care	#30	Provisions, if any, for ancillary and post-trial care, and for compensation to those who suffer harm from trial participation	N/A none
Dissemination policy: trial results	#31a	Plans for investigators and sponsor to communicate trial results to participants, healthcare professionals, the public, and other relevant groups (eg, via publication, reporting in results databases, or other data sharing arrangements), including any publication restrictions	22
Dissemination policy: authorship	#31b	Authorship eligibility guidelines and any intended use of professional writers	22
Dissemination policy: reproducible research	#31c	Plans, if any, for granting public access to the full protocol, participant-level dataset, and statistical code	N/A none
Informed consent materials	#32	Model consent form and other related documentation given to participants and authorised surrogates	20
Biological specimens	#33	Plans for collection, laboratory evaluation, and storage of biological specimens for genetic or molecular analysis in the current trial and for future use in ancillary studies, if applicable	N/A

Author notes

1. N/A - no moficiations

The SPIRIT checklist is distributed under the terms of the Creative Commons Attribution License CC-BY-ND 3.0. This checklist was completed on 26. June 2018 using http://www.goodreports.org/, a tool made by the EQUATOR Network in collaboration with Penelope.ai

For peer review only

BMJ Open

Clinical and Cost-effectiveness of One Session Treatment (OST) vs. Multi-session Cognitive Behaviour Therapy (CBT) for Specific Phobias in Children: Protocol for a Non-inferiority Randomised Controlled Trial

Journal:	*BMJ Open*
Manuscript ID	bmjopen-2018-025031.R1
Article Type:	Protocol
Date Submitted by the Author:	13-Jul-2018

Complete List of Authors:	Wright, Barry; Lime Trees CAMHS,<br>Cooper, Cindy<br>Scott, Alex; Sheffield University, School of Health and Related Research<br>tindall, Lucy<br>Ali, Shehzad; University of York, Department of Health Sciences<br>Bee, Penny; University of Manchester,<br>Biggs, Katie; University of Sheffield, Clinical Trials Unit<br>Breckman, Trilby<br>Davis III, Thompson; Louisiana State University College of Humanities and Social Sciences, Psychological Services Center<br>Gega, L; University of York<br>Hargate, Rebecca; Lime Trees CAMHS Centre, Research<br>Lee, Ellen; University of Sheffield, Clinical Trials Research Unit<br>Lovell, Karina; The University of Manchester, The School of Nursing, Midwifery and Social Work<br>Marshall, David; Lime Trees CAMHS , Research Team<br>McMillan, Dean; University of York, Department of Health Sciences<br>Teare, M; University of Sheffield, School of Health and Related Research<br>Wilson, Jon

**Primary Subject Heading**:	Research methods
Secondary Subject Heading:	Mental health, Paediatrics
Keywords:	Phobia, Children and adolescents, randomised controlled trial, one-session treatment, exposure therapy

SCHOLARONE™
Manuscripts

ALLEVIATING SPECIFIC PHOBIAS EXPERIENCED BY CHILDREN TRIAL (ASPECT)

Clinical and Cost-effectiveness of One Session Treatment (OST) vs. Multi-session Cognitive Behaviour Therapy (CBT) for Specific Phobias in Children: Protocol for a Non-inferiority Randomised Controlled Trial

Barry Wright[1], Cindy Cooper[2] , Alexander J Scott[2], Lucy Tindall[1], Shehzad Ali[3] , Penny Bee[4], Katie Biggs[2], Trilby Breckman[5], Thompson Davis[6], Lina Gega[3], Rebecca Hargate[1*], Ellen Lee[2] , Karina Lovell[4], David Marshall[7], Dean McMillan[3], Dawn Teare[2], and Jonathan Wilson[8]

Word count: 5940

* Corresponding author Rebecca.hargate@nhs.net

[1]Child Oriented Mental Health Intervention Centre, IT Centre, Innovation Way, Heslington, York, YO10 5NP, UK

[2]ScHARR, The University of Sheffield, Regent Court, 30 Regent Street, Sheffield, S1 4DA, UK

[3] Department of Health Sciences & Hull York Medical School, University of York, Heslington, York, YO10 5DD, UK

[4] Division of Nursing, Midwifery and Social Work, University of Manchester, Oxford Road, Manchester. M13 9PL, UK

[5] Triumph Over Phobia (TOP UK), PO Box 3760, Bath, BA2 3WY, UK

[6] Psychological Services Center, Louisiana State University, Baton Rouge, LA, 70803, USA

[7] Centre for Reviews and Dissemination, University of York, Heslington, York, YO10 5DD, UK

[8] Norfolk and Suffolk NHS Foundation Trust, 80 St Stephens Rd, Norwich, NR1 3RE, UK

For peer review only - http://bmjopen.bmj.com/site/about/guidelines.xhtml

ALLEVIATING SPECIFIC PHOBIAS EXPERIENCED BY CHILDREN TRIAL (ASPECT)

Abstract

Introduction: Specific phobias (intense, enduring fears of an object or situation that lead to avoidance and severe distress) are highly prevalent among children and young people. Cognitive Behaviour Therapy (CBT) is a well-established, effective intervention, but it can be time-consuming and costly because it is routinely delivered over multiple sessions during several months. Alternative methods of treating severe and debilitating phobias in children are needed, like One-Session Treatment (OST), not only to reduce time and cost, but also to prevent therapeutic drift and help children recover quickly. Our study explores whether (a) outcomes with OST are 'no worse' than outcomes with multi-session CBT, (b) OST is acceptable to children, their parents and the practitioners who use it, and (c) OST offers good value for money to the National Health Service (NHS) and to society. **Method**: A pragmatic, non-inferiority, randomised controlled trial (RCT) will compare OST with multi-session CBT based therapy on their clinical and cost-effectiveness. The primary clinical outcome is a standardised behavioural task of approaching the feared stimulus at six-months post randomisation. The outcomes for the within-trial cost-effectiveness analysis are quality-adjusted life years (QALYs) based on EQ-5D-Y, and individual-level costs based of the intervention and use of health and social service care. A nested qualitative evaluation will explore children's, parents' and practitioners' perceptions and experiences of OST. A total of 286 children, 7-16 years old, with DSM-IV diagnoses of specific phobia will be recruited via gatekeepers in the NHS, schools and voluntary youth services, and via public adverts. **Ethics and dissemination**: The trial received ethical approval from North East and York Research Ethics Committee (Reference: 17/NE/0012). Dissemination plans include publications in peer-reviewed journals, presentations in relevant research conferences, local research symposia, and seminars for children and their families, and for professionals and service managers. **Trial Registration Number:** ISRCTN19883421.

For peer review only - http://bmjopen.bmj.com/site/about/guidelines.xhtml

ALLEVIATING SPECIFIC PHOBIAS EXPERIENCED BY CHILDREN TRIAL (ASPECT)

Keywords: phobia; children and adolescents; randomised controlled trial; one-session treatment; exposure therapy.

For peer review only

For peer review only - http://bmjopen.bmj.com/site/about/guidelines.xhtml

Strengths & Limitations

- To our knowledge, this is the largest proposed treatment study and economic evaluation of interventions for childhood phobias to-date.
- Interventions are delivered by multi-disciplinary professionals working in multiple sectors including health, education, and voluntary organisations.
- Children with all types of specific phobias and different learning abilities are included.
- There are no mid-point outcomes; therefore, the study cannot tell whether children improve with one-session treatment quicker than with multi-session therapy or how the two interventions may work in different ways to change outcomes (mechanisms of action). However, the study includes longer-term outcomes which are useful in evaluating sustained effect, if any, of the intervention.

For peer review only

ALLEVIATING SPECIFIC PHOBIAS EXPERIENCED BY CHILDREN TRIAL (ASPECT)

Introduction

A specific phobia is an intense, enduring fear of an identifiable object or situation that leads to distress and avoidance[1]. It is estimated that between 5% and 10% of children have a specific phobia severe enough to impact on their everyday functioning[2], but fewer than 10% seek treatment, therefore the average duration of phobias is 20 years[3]. The psychological, developmental and medical impact of these phobias is significant, with higher rates of health service usage than most other anxiety disorders[4]. Phobias in children can result in considerable academic difficulties[5], personal distress[6] and interference in day-to-day activities[7]. Cognitive Behavioural Therapy (CBT) is the dominant therapeutic intervention for specific phobias[8 9] within health services in the UK. Robust evidence supports the efficacy of CBT for anxiety disorders in general [10-12], and for specific phobias in particular; however, CBT can be time-consuming, costly, and difficult to access because it is delivered over multiple sessions during several weeks or months by therapists that have received lengthy training [13-16].

Alternative methods of CBT delivery, like One-Session Treatment (OST)[17], can be used to reduce demands on therapist time and associated costs, prevent therapeutic drift, and ultimately help children recover quicker [18 19]. OST is a variant of CBT, delivered in a single three-hour session. A central feature is graded exposure where the subject comes into proximity with the phobic stimulus in a series of graduated steps (from the easiest to the most difficult). For example, graded exposure for a phobia of dogs might first involve looking at pictures of dogs, followed by videos of dogs, then by observing dogs in a local park at a distance, and finally by interacting with dogs first hand. OST has been shown to be clinically effective in children[20-22]. For example, Ollendick and colleagues[20] randomised 196 children (ages 7 to 16) with phobias to one of three groups: (i) One Session Treatment; (ii) an education support group; or (iii) a wait list control group. OST was superior to both

For peer review only - http://bmjopen.bmj.com/site/about/guidelines.xhtml

comparators as measured by improved phobia severity, percentage of children who were diagnosis free, and child- and parent-rated anxiety and treatment satisfaction post-intervention and at six months follow-up. However, to our knowledge, OST has not been compared to the current 'gold-standard' approach to specific phobias (i.e., CBT), and has not been trialled in the UK within the National Health Service (NHS).

The present research is a non-inferiority, parallel group randomised controlled trial comparing the clinical and cost-effectiveness of OST vs. CBT for specific phobias in children and young people ages 7 to 16 years old. The primary aim is to test whether OST is non-inferior (i.e., produces *similar* clinical benefits) to multi-session CBT in helping children with their specific phobia(s), and the wider impact their phobia(s) have on mental health and quality of life. A nested qualitative evaluation will explore whether OST is acceptable to children, their parents, and the practitioners who deliver OST. Finally, an economic evaluation will examine whether OST offers good value for money to the NHS and society compared to multi-session CBT.

Methods and Analysis

Participants

The present research will recruit children and young people aged 7-16 years old with one or more specific phobias, as defined by the DSM-IV[1]. The DSM-IV criteria are: i) marked and out-of-proportion fear to a specific object or situation, ii) exposure to the feared object or situation provokes immediate anxiety, iii) the phobic situation is avoided where possible, iv) the avoidance or distress associated with the phobia interferes with the person's routine or functioning (e.g. learning, sleep, social activities) and v) the phobic avoidance or distress is present for 6 months or more. We include all types of phobias where we can introduce or safely simulate the stimulus for the purpose of exposure therapy. We exclude children who have conditions that are often referred to as "phobias" but are not *specific*

For peer review only - http://bmjopen.bmj.com/site/about/guidelines.xhtml

phobias (e.g. social or separation anxiety referred to in some circumstances as "school phobia") or when children need a different intervention as a priority (e.g. for psychosis or self-harm).

Design

This is a non-inferiority randomised controlled trial that compares clinical outcomes of OST with those of multi session CBT at 6 months post-randomisation. Nested within this trial is an economic evaluation of the cost-effectiveness of OST against CBT for outcomes and costs over 6 months. Interviews evaluate perceptions and experiences of OST by children, parents and practitioners also at 6 month follow-up. An internal 9-month pilot determines feasibility of recruitment and retention, and monitors the occurrence of any adverse events.

Recruitment

Our recruitment strategies follow routine care pathways for children with specific phobias. These pathways differ across our participating sites, which include NHS Trusts, Local Authorities and voluntary agencies across England. We have mapped the care pathways in each geographical area and we promote the study within teams of professionals who routinely receive referrals of children with specific phobias, such as Child and Adolescent Mental Health Services (CAMHS), Children's Hospitals, youth services and schools. We also draw attention of the study to potential participants through public adverts, e.g. in local newspapers and posters in GP surgeries.

Randomisation

Following written consent and completion of the baseline measures, random allocation to treatment groups is carried out remotely through a secure web-based program designed by the Sheffield Clinical Trials Unit (CTRU). The randomisation allocation ratio is 1:1 to facilitate equal sample sizes across both the OST and CBT groups. Randomisation is

For peer review only - http://bmjopen.bmj.com/site/about/guidelines.xhtml

stratified according to two age groups (7-11 years old vs. 12-16 years old) and two phobia severity groups (mild-to-moderate vs. severe); we use random permuted blocks of variable size to ensure enough participants are allocated evenly to each arm of the trial within each stratum.

Blinding

Complete concealment of allocation, “blinding”, from both participants and researchers in studies of psychological interventions is often extremely difficult, and, at times, impossible[23-26]. Our study takes a number of steps to facilitate blind assessment of outcomes as a way of reducing bias. First, the research assistants collecting baseline and follow-up outcomes are independent of the practitioners delivering the interventions. Second, the research assistants are not informed of the participants’ group allocation and are not involved in the therapy sessions. Participants are explicitly reminded not to disclose to the research assistant their allocated group. In the event of these procedures being compromised (e.g. a research assistant learning the participant’s allocated group), we record this and arrange for a different outcome assessor in the future wherever possible. The trial statisticians and health economists are blind to treatment allocation whilst the trial is ongoing.

Interventions

One-Session Treatment (OST)

OST is a variant of CBT, but rather than being delivered in weekly 45-60 min sessions as in conventional CBT, OST is delivered in a longer single session (approximately 3 hours). Prior to the single session, a 1-hour assessment/planning session takes place during which the practitioner carries out an assessment in order to plan the treatment session. This involves:

a) determining the nature, history and associations of the phobia.

For peer review only - http://bmjopen.bmj.com/site/about/guidelines.xhtml

b) determining any maintaining factors for the phobia (e.g. what the child avoids, what safety behaviours s/he engages in, how friends and family may collude with the phobia);

c) collecting information about the child's cognitions including catastrophic thoughts (e.g. what do you think may happen if…, what goes through your mind when you…);

d) generating a fear hierarchy (i.e. a 'ladder' of situations or objects that the child avoids because of their phobia, in the order of the fear or anxiety evoked, starting from the least frightening).

e) explaining what is involved in treatment and that the exposure tasks are graded and negotiated in every step of the way and that nothing will ever happen without the child's permission.

The single session combines graded exposure, participant modelling, reinforcement, and cognitive challenges. The main principle of graded exposure is that the child gradually confronts the situations from their fear hierarchy and remains in each situation until their anxiety/fear subsides at least by 50%. In participant modelling, the therapist first demonstrates to the child how to interact with the phobic object and then helps the child gradually approach the phobic object by joint approaches with the therapist gradually fading involvement. Reinforcement from the therapist may take the form of social praise, encouragement etc. Finally, the therapist uses the exposure tasks as means for actively eliciting, challenging, and testing negative cognitions or catastrophic thoughts associated with the feared situation and by promoting positive cognitions in phobic situations. It is important that the child maximises, maintains and generalises their gains from graded exposure by practising self-directed and parent/carer supported exposure tasks at home for the ensuing weeks. Maintenance tasks include a commitment to refrain from avoiding or escaping from feared situations and to deliberately engineer or enter a feared situation regularly.

For peer review only - http://bmjopen.bmj.com/site/about/guidelines.xhtml

Cognitive Behavioural Therapy (CBT)

CBT helps people to change unhelpful beliefs and behaviours arising in feared situations, such as catastrophic thinking and safety behaviours. Interventions based on CBT are the dominant model of treatment for specific phobias, and are supported by a robust literature attesting to its efficacy [8-12]. CBT interventions are often delivered in 45-60 minute sessions every week or fortnight over several weeks or months. Each CBT session takes the form of structured discussions with a therapist, has a specific agenda, and sets homework tasks for the child or young person between sessions. CBT for children and young people with specific phobias aims to help the child/young person to: (i) recognise anxious feelings and bodily reactions to anxiety; (ii) gradually confront their feared situations until their anxiety subsides; (iii) capture and challenge anxious thoughts when faced with a phobic situation or object; (iv) challenge avoidance behaviours, or behaviours that maintain or reinforce the phobia; and (v) develop coping strategies and anxiety management techniques if distress and physical responses to anxiety are overwhelming. There is no recommended number of CBT sessions for specific phobias but it can vary between 4 and 20 sessions.

Fidelity to the interventions

Practitioners responsible for specific phobia treatment across the recruiting centres will be trained in OST during a day's workshop, with senior practitioners (e.g. trained therapists) attending a modified 1/2 –day training on OST and a ½-day training on supervising and training others in OST to be able to offer continuous support and supervision to junior colleagues. OST and CBT sessions are audio-recorded whenever possible with consent from the child and parent. A random selection of 15% of participants at each site (or the nearest whole number) has one of their sessions (whether OST or CBT) reviewed by a supervisor at quarterly intervals. Supervisors score the recorded sessions to assess therapist fidelity to OST and CBT using the OST Rating Scale (OST-RS)[27] and the Cognitive

For peer review only - http://bmjopen.bmj.com/site/about/guidelines.xhtml

Behaviour Therapy Scale for Children and Young People CBTS-CYP, [28] respectively. This is to make sure that CBT principles are applied in both treatment arms. These are used in supervision to improve fidelity to the intervention and address training needs for the therapists as per usual practice.

Outcome Measures

We are collecting demographic information (e.g. age, gender etc.) from all children/young people and their parents. Children and parents are also asked if they have a treatment preference at baseline, although this does not affect their randomisation in any way. Participants complete a series of questionnaires at baseline and then at 6 months follow up, as described below.

Behavioural Avoidance Task (BAT)

The primary outcome is the BAT at six-months post randomisation. The BAT is a widely used measure of treatment outcomes for phobias in children[6 21]. The BATs for the ASPECT trial are standardised for each stimulus and have been found to have good test-retest reliability[20 29] and strong correlation with other outcome measures of anxiety and phobia[30 31]. During a BAT, the child gradually confronts their phobic stimulus over ten pre-defined steps of increasing difficulty; the child does not have to complete all the steps. For example, a child with spider phobia may start the BAT at step 0, standing outside of a room that has a spider contained in a box. Subsequent steps the child may take are: opening the door (step 1), passing through the doorway (step 2), through to holding the spider for 20 seconds (step 10). The number of steps the participant takes is the main unit of measurement recorded for analysis. The BAT also includes a measure of subjective units of distress (SUDs) whereby the child reports their level of fear at the start of the BAT, and at the last step completed (ranging from 0 = no fear at all to 8 = extreme fear).

For peer review only - http://bmjopen.bmj.com/site/about/guidelines.xhtml

The Anxiety Disorder Interview Schedule for Children and Parents (ADIS-C/P)

ADIS; [32] is a well-established semi-structured interview[3 32 33] that obtains information from both the child and their parent/carer about the type of specific phobias experienced (e.g. animal/insect, small spaces, blood/injection phobias etc.), the degree of associated fear (rated from 0=not at all to 8=very, very much),whether the phobia causes avoidance (rated as 'yes' or 'no'), and interference with daily life (rated from 0=not at all to 8=very, very much).

Child Anxiety Impact Scale for Children and Parents (CAIS-C/P)

The CAIS-C/P; [34] collects information as to what extent feeling 'nervous, anxious or afraid' impacts on the daily life of the child. The child and the parent independently rate, on a 4-point scale ranging from 0=not at all to 4=very much, 27 statements grouped in 3 sub-domains: school activities (e.g. *getting to school on time in the morning*), social life (e.g. *making new friends*) and home/family life (e.g. *getting ready for bed at night*).

The Revised Children's Anxiety and Depression Scale (RCADS-C/P)

The RCADS-C/P[35-38] comprises 47 items to capture separation anxiety disorder (SAD), social phobia (SP), generalized anxiety disorder (GAD), panic disorder (PD), obsessive compulsive disorder (OCD), and major depressive disorder (MDD). Children state the frequency of which statements such as "*I worry about things*", *I have trouble sleeping*" and "*I feel I will make a fool of myself in front of people*" relate to them on a 4-point scale: 0=never, through to 1=sometimes, 2=often, and 3=always.

EQ-5D-Y

The EQ-5D-Y, originally developed by the EuroQoL group[39 40 41], is a widely used measure of health-related quality of life in children and young people. Children and young people are able to classify their own health on a three-point scale, 1=no problems, 2=some problems and 3=a lot of problems, over five dimensions: mobility (walking about), looking after myself, doing unusual activities, having pain or discomfort, and feeling worried, sad or

For peer review only - http://bmjopen.bmj.com/site/about/guidelines.xhtml

unhappy. Additionally, the EQ-5D-Y includes a visual analogue scale where participants can indicate their overall health status from 0 (worst imaginable state) to 100 (best imaginable state). All questions refer to the participant's health state "today".

Child Health Utility 9D (CHU-9D)

The CHU-9D is a health related quality of life measure validated for children and young people[42 43]. Participants select a sentence from a possible five to describe how they feel today in relation to a number of constructs (i.e. sadness, tiredness, pain, etc.). For example, participants are asked to select one of five statements that best reflects how worried they feel ranging from "*I don't feel worried today*" to "*I feel very worried today*".

Goal-based Outcome Measure

A goal-based outcome measure is a method of comparing how far a participant feels they have moved towards reaching a specific goal they have set before the intervention has begun. A goal-based outcome measure based on recent guidelines[44] will be used to set up to three goals at baseline, with progress towards meeting these goals rated on a 10-point scale at the 6-month follow-up point. Progress can range from 0 (i.e. goal not met), to 5 (i.e. half-way to reaching the goal), through to 10 (i.e. goal reached). For example, a participant with a dog phobia may set as their goal that they would like to be able to spend teatime at their grandmother's house where there is a dog.

Resource Utilisation Questionnaire

We developed a bespoke questionnaire to assess use of healthcare and other resources, based on a previous resource utilisation questionnaire by Barrett and colleagues[45] and its adaptations for younger populations by members of our research team [45 46 47]. The questionnaire is completed by the parent and collects data on resource and service utilisation relating to the child over the previous 6 months on: general health and community service use (i.e. appointments with GP's, nurses, social services and educational services); mental health

For peer review only - http://bmjopen.bmj.com/site/about/guidelines.xhtml

service use (i.e. appointments with a psychiatrist, psychotherapists, psychologists, CAMHS therapists and other forms of mental health support); hospital-based services (i.e. visits to A & E, urgent care centres and hospital stays); days missed from school by the child; days missed by parents/carers from work or studies.

Statistical Analysis

Sample Size

The proposed sample size and non-inferiority margin are based on two separate Cochrane reviews looking at the effects of psychotherapy for those experiencing anxiety. First, Wolitzky-Taylor and colleagues[47] conducted a review on studies that used both observer-rated BAT-like assessments and self-report questionnaires on adults with specific phobias and reported an overall, large effect size of $d = 0.81$. However, as the treatment may have a different effect on children, we also examined the review of Reynolds and colleagues[48]. This review was conducted on studies of children with specific phobias but used self-report questionnaires rather than observer-rated BATs. The review reported a large effect size ($d = 0.85$) for multi-session CBT. Prior meta-analyses suggest that a standardised mean difference of around 0.8 on the BAT scale is clinically important. Therefore, we set the non-inferiority margin to be half of this at 0.4[49]. Assuming a correlation of 0.5 between baseline and final BAT measure, we would require 200 participants (100 in each arm) to have 90% power with a 2.5% one-sided significance level to demonstrate non-inferiority of OST compared to CBT. The therapy is delivered by therapists who will see approximately 15 patients each and we anticipate a weak therapist effect (intraclass correlation coefficient (ICC = 0.01)). This clustering will lead to a design effect of 1.14 which increases the number required per arm to 114. We further assume a 20% dropout rate suggesting that total number of 286 children and young people (143 per arm) will need to be recruited to the study to demonstrate non-inferiority of OST compared to CBT.

For peer review only - http://bmjopen.bmj.com/site/about/guidelines.xhtml

Data Analysis

As this trial follows a randomised, parallel group, non-inferiority design, data will be analysed and reported according to both CONSORT guidelines[50] and the non-inferiority trials CONSORT extension[51]. Baseline data, including demographic characteristics (e.g. age, gender) and clinical measures (e.g. BAT, RCADS), will be reported between the two randomised groups. Jones and colleagues[49] recommend both per protocol and intention to treat (ITT) analyses for non-inferiority designs. For an equivalence or non-inferiority trial, as opposed to a comparative trial, ITT analyses are not considered conservative, as any blurring of the difference between the treatment groups increases the chance of declaring equivalence. We follow this recommendation with the refinement that the main analysis of the primary outcome will be per protocol (or completers only) with a sensitivity analysis on the ITT population[49]. We will require both the per protocol and ITT analyses to demonstrate conclusive evidence to declare that OST is "non-inferior" to multi-session CBT. If the results of the analysis are discrepant (e.g. the ITT rejects the null of inferiority but the per protocol analysis does not, or vice versa) then we will report the conflicting results from both analyses highlighting the inconclusive nature of the results. For the sake of the analysis a participant in the OST group is defined as per protocol if they attend both the initial functional assessment and the rapid exposure therapy session. A participant in the CBT group is defined as per protocol if they attend at least 4 CBT sessions.

The primary outcome (mean BAT score at 6 months) and the secondary outcomes (ADIS, CAIS, EQ-5D-Y, CHU-9D, RCADS and goal-based outcome scores) will be compared between groups using mixed effects linear regression with exchangeable correlation to allow for the clustering of outcomes within therapist. The analysis will be conducted controlling for baseline BAT score, site, and stratifying variables (age group and baseline phobia severity). The null hypothesis of inferiority will be rejected if the two sided

For peer review only - http://bmjopen.bmj.com/site/about/guidelines.xhtml

95% confidence interval (CI) for the difference is wholly below 0.4 (the range of clinical non-inferiority).

We anticipate some dropout/attrition, so case missing data may be an issue. Case and item missing data will be examined and multiple imputation methods will be used to reduce bias due to any missing responses in both the per protocol and the ITT analyses. Where appropriate, modelling methods that generate robust standard errors in the presence of missing data will be considered.

Economic Evaluation

We are conducting an economic evaluation from the UK personal and social services perspective. This will take the form of a within-trial cost-utility analysis to determine the incremental cost per unit of quality-adjusted life years (QALYs) of OST compared with CBT in children with specific phobias. Costs will be calculated based on: i) resources required to deliver the intervention and ii) individual-level use of health and social services by children and absenteeism from school for children or from work because of childcare for parents over the study period. Resource use will be multiplied by unit costs to arrive at total cost in each arm. QALYs will be calculated by measuring health-related quality of life using the self-reported EQ-5D-Y over the study period.

The resources required to deliver the OST intervention will be calculated using bottom-up estimation of the time spent by professionals as well as other resources used (including phobic stimulus acquisition, i.e. animal hire). Individual-level service use data will be based on self-reported use of primary and secondary health care as well as social care using a standardised resource utilisation questionnaire. Unit costs of health and social service use will be obtained from the UK national database of reference costs Department of Health, [52] and the Unit Costs of Health and Social Care report produced by the Personal Social

For peer review only - http://bmjopen.bmj.com/site/about/guidelines.xhtml

Services Research Unit[53]. The cost of medication will be based on the most recent version of the British National Formulary (BNF)Royal Pharmaceutical Society of Great Britain, [54].

Individual-level responses on the EQ-5D-Y will be used to estimate health related quality of life based on a UK population valuation set. Subsequently, an area under the curve approach will be used to calculate QALYs for each child. QALY is a utility-based measure, i.e. it measures each person's health state in terms of quality of life dimensions and then weights it on the value or utility of the health state based on UK population preferences[55].

The primary economic analysis will be cost-utility analysis conducted over the trial follow-up period (6 months). Total costs, including the intervention cost and service utilisation costs, and QALYs will be compared between the two interventions. Unadjusted costs and outcomes will be presented in a descriptive analysis using parametric and non-parametric tests. The statistical analysis will compare mean costs and QALYs using a regression model. The regression analysis will control for baseline differences in utility[56]. The specification of the model will follow the approach recommended by Glick, Doshi, Sonnad, and Polsky[57] which considers the distribution of the dependent variable as well as any correlation between the cost and QALY outcomes. The regression coefficient on treatment will then represent the difference in mean cost and mean QALYs between groups. A bootstrap method will be used to produce confidence intervals around the cost and QALY differences due to the likely skewness in the distribution of regression residuals[58]. To present this in the UK decision-making context, the results will be in the conventional form of a cost-effectiveness acceptability curve (CEAC). CEAC presents the probability of the intervention being cost-effective over a range of willingness-to-pay (WTP) thresholds per QALY[59]. The higher the probability, the more likely it is that the treatment is cost-effective at the particular WTP threshold.

For peer review only - http://bmjopen.bmj.com/site/about/guidelines.xhtml

The following sensitivity analyses will be conducted; i) a cost-utility analysis using the CHU9D instrument instead of EQ-5D; ii) a cost-effectiveness analysis using a phobia-specific measure instead of a utility-based measure; and iii) cost-utility analysis from a health services perspective.

Nested Qualitative Evaluation

We will invite a sub-sample of trial participants to participate in individual semi-structured interviews and use maximum variation sampling to ensure a spread of participants differing in age, gender, socio-economic background and type of phobia. We will obtain written consent (and assent where appropriate) to interview a sample of i) children receiving OST, ii) their parents/guardians and iii) practitioners delivering OST across all study sites. With parental consent. We will recruit and interview parents and children separately. Joint parent-child interviews will be included where participants prefer. The final sample size for the interviews in each participant group will be determined by data saturation, i.e. the point where no new themes, ideas and/or concepts emerge. Based on a previous nested qualitative evaluation of patient acceptability of brief psychological interventions[60], we estimate that we will need to complete a maximum of 30 interviews with parents, 25 with children and 15 with practitioners.

Interviews with children and their parents will be conducted after participants have completed the final outcome measures at the 6-month follow-up point. Interviews for parents will focus on phobia experiences, personal and family impact, perceived treatment need, treatment expectations and treatment acceptability (e.g. content, delivery mode, format, setting and facilitation). Child interviews will focus on the same topics, adapted for age and developmental maturity. Face-to-face interviews with children will draw on the principles of 'draw and write' techniques, whereby children will be offered an opportunity to draw a picture relating to their experiences as a prompt to initiate more in-depth discussion[61 62].

For peer review only - http://bmjopen.bmj.com/site/about/guidelines.xhtml

Child interviews will last a maximum of 30 minutes and parent interviews a maximum of 60 minutes, with total time guided by the interviewees. Clinician interviews will last for a maximum of 60 minutes and focus on their experiences and views of delivering OST, the individual and organisational support required and the perceived suitability OST for their patient group.

Interviews with parents and older children (13 years plus) will be conducted face-to-face in treatment settings or at participants' homes, or via the telephone, depending upon participant preference. Interviews with younger children (12 years and under) will be conducted face-to-face in treatment settings or at participants' homes, depending upon participant preference. Practitioner interviews will take place face-to-face in their workplace or over the telephone once their own involvement in the trial is complete.

All interviews will be digitally recorded and transcribed verbatim. Analysis will follow a qualitative framework approach[63], a widely used method of analysing primary qualitative data pertaining to health care practices with policy relevance[64]. Framework analysis permits both deductive and inductive coding, enabling potentially important themes or concepts that have been identified a priori to be combined with additional themes emerging de novo.

Data coding will be undertaken independently by two trained researchers. We will additionally train a Patient and Public Involvement (PPI) representative to code alongside these researchers to ensure coding takes account of potential differences in perspective. Coders will meet regularly to develop a shared coding manual and to ensure that all emerging codes remain grounded in original data. An Excel spread sheet will be developed which will incorporate preliminary framework themes as column headings and the demographic information related to participants who provided data under each theme. As the constant comparison of new data occurs and the coding team's understandings of the themes under

For peer review only - http://bmjopen.bmj.com/site/about/guidelines.xhtml

consideration develop, the framework will be amended and re-shaped to enable the introduction of new codes and/or the deletion of redundant, similar or otherwise compromised codes. In this way, a final framework will be achieved that is considered representative of the entire dataset. We will code data from each stakeholder group (children, parents/guardians and practitioners) separately before comparing and contrasting data findings across groups. The final coding manuals for each participant group will be presented with example entries, to the project's advisory committees to confirm validity, coherence and conceptual relevance.

Public and Patient Involvement

The research protocol was developed in partnership with service user groups and was reviewed in terms of both feasibility and relevance. In addition to this, the views of relevant charitable organisations were sought and a representative from the Triumph Over Phobia UK support group is a co-applicant on this application. We have consulted with PPI representatives about their views on the full research proposal and how best to involve patients throughout the proposed project. In particular, we have discussed comments after the first round application with representatives of Triumph Over phobia. This includes issues around the non-inferiority design, how to measure fidelity and contamination. We have also engaged the York Youth Council, who have commented on this application and discussed the methodology of the trial and the potential impact of the therapy on the community. We see PPI as central to our research. A representative from Triumph Over Phobia are on our trial management group and actively involved in the planning and delivery of this research. Applicants KL and PB are named applicants (PI-and Co-PI respectively) on an NIHR-funded programme grant seeking to improve user involvement in mental health care planning. This programme has been recognised by the MHRN for exemplary carer involvement. Applicant

For peer review only - http://bmjopen.bmj.com/site/about/guidelines.xhtml

PB has substantial experience of training users and carers in research methodologies, interviewing skills and qualitative data analysis. We recognise the need for independent qualitative data analysis, and will train a service user researcher. The service user representative will be reimbursed for their time, commensurate with current INVOLVE guidelines.

Ethics and dissemination

Obtaining informed consent from participants

Written informed consent from children and parents is taken at a face-to-face meeting with a research assistant. As all participants in this study are aged 16 or under, consent is required from both a person with parental responsibility, and the participant themselves if they are deemed competent to do so. Where a child is not deemed competent to give full informed consent, we take parental consent and seek assent from the child. Prior to the consent meeting, the parents and the children receive information about the study and have the opportunity to discuss any questions or concerns with members of the research team. Two age-specific versions of the child information sheets are available: one for those aged 7 to 11 years and one for those aged 12 to 16 years. All information leaflets and consent forms are co-developed by the research team and PPI representatives to ensure acceptability amongst participants. If a participant wishes to withdraw from their allocated intervention, we check whether they are still agreeable to participate in the planned follow-up assessment at 6 months, so that their outcomes can be included within our ITT analysis.

Data management

Trial data are extracted from source documents and entered onto the CTRU's in-house data management system (Prospect). Prospect stores data in a PostgreSQL database hosted by the University of Sheffield. The database uses industry standard techniques to provide security, including password authentication and encryption using SSL/TLS. Access to

For peer review only - http://bmjopen.bmj.com/site/about/guidelines.xhtml

Prospect is controlled by usernames and encrypted passwords. A comprehensive privilege management feature ensures only the minimum amount of data required is available to each individual to complete their tasks. The system has a full electronic audit trail and is regularly backed up. Access to data is restricted to users with appropriate privileges only. All data are collected and retained in accordance with the Data Protection Act 1998 and Sheffield Clinical Trial Research Unit (CTRU) standard operating procedures (SOPs).

Research governance and conduct of the trial

Trial monitoring procedures and site monitoring are undertaken at a level appropriate to a risk assessment performed by the Trial Sponsor (Leeds and York Partnership NHS Foundation Trust, 2150 Century Way, Thorpe Park, Leeds, LS15 8ZB) and the Sheffield Clinical Trial Research Unit (CTRU). Three committees govern the conduct of this study, in accordance with Sheffield CTRU's Standard Operating Procedures (SOPs): a Trial Steering Committee (TSC), an independent Data Monitoring and Ethics Committee (DMEC) and a Trial Management Group (TMG)

The TSC consists of an independent Chair, an independent subject specialist, an independent clinical academic, an independent statistician and a patient representative. The TSC meets approximately every 6 months from the start of the trial. The DMEC consists of an independent chair, an independent statistician, and an independent ethics specialist experienced in research with children and families. The TMG comprises the co-applicants and the two trial managers who are jointly supervised by the CI, the Director of the Sheffield CTRU and a lead trial manager in the CTRU. Meeting attendance of the co-applicants depends on each meeting's agenda and relevance to their role.

Adverse events

An adverse events monitoring form is used to record any untoward occurrence affecting the participant after each therapy session by the therapist and at follow-up by the

For peer review only - http://bmjopen.bmj.com/site/about/guidelines.xhtml

research assistant. Such an event can be directly related, possibly related or unrelated to the intervention. An occurrence is recorded if it is suspected to be related to the intervention or an aspect of the research procedures; the therapist assesses relatedness and research assistants seek advice from the site Principal Investigator. The occurrence of adverse events during the trial is monitored by the trial oversight and management groups, particularly the DMEC and the TSC. All adverse events are assessed for seriousness, and will be recorded as a Serious Adverse Event (SAE) if they are life-threatening, or require hospitalisation or prolongation of existing inpatient stay, or result in persistent or significant disability or incapacity or death.

Ethical approval

This study has received ethical approval from the North East - York Research Ethics Committee (Reference: 17/NE/0012). No pharmaceutical compounds or medical devices are used in this trial, therefore Clinical Trials Authorisation is not required.

Dissemination of the research findings

We will publish the study's findings in peer reviewed academic journals and present at local, national, and international conferences where possible. Furthermore, we will publish a short summary of the results on the ASPECT study website that can be accessed by all trial participants as well as relevant interest groups, including patient groups. Finally, towards the end of the trial, our Patient and Public Involvement (PPI) representatives will organize a meeting with stakeholders including parents and professionals working with young people with anxiety disorders to specifically discuss the dissemination of the study findings and put together a dissemination plan.

Trial status

Protocol V3 24/04/17. Recruitment to the trial began in June 2017 and is estimated to be completed in July 2019, with the final follow up assessments completed in March 2020.

For peer review only - http://bmjopen.bmj.com/site/about/guidelines.xhtml

Author Contributions

BW, DM, TD, LT, LG,DMc, KL,PB, RH & CC designed the study and drafted the original grant proposal BW & CC provided trial leadership. CC, KB & RH provide governance oversight. CC, BW, DM, DMc, DT, KL, TD provide methodological expertise. SA provides health economics expertise. DT & EL provide statistical and data management expertise. LG, DMc, KL, JW, and BW provide clinical expertise. TD, BW and LG provide training and supervision in OST and CBT. PB provides qualitative expertise. AS & LT are the trial's managers. TB leads on public and service user representation. All authors contributed to the writing and review of the trial's protocol. All co-authors approved the final version of this manuscript.

Funding

This article presents independent research funded by the National Institute for Health Research (NIHR) under its Health Technology Assessment (HTA) Programme (Grant Reference Number HTA15/38/04). The views expressed are those of the author(s) and not necessarily those of the NHS, the NIHR or the Department of Health.

Competing Interests

Dr Thompson Davis receives royalties from the sale of a book on OST. There are no competing interests for the other authors.

For peer review only

References

1. Association AP, Association AP. DSM-IV-TR: Diagnostic and statistical manual of mental disorders, text revision. *Washington, DC: American Psychiatric Association* 2000;75:78-85.

2. Kessler R, Berglund P, Demler O, et al. Lifetime prevalence and age-of-onset distributions of DSM-IV disorders in the national comorbidity survey replication. *Archives of General Psychiatry* 2005;62:593-602.

3. Stinson FS, Dawson DA, Chou SP, et al. The epidemiology of DSM-IV specific phobia in the USA: results from the National Epidemiologic Survey on Alcohol and Related Conditions. *Psychological Medicine* 2007;37(7):1047-59. doi: 10.1017/s0033291707000086

4. Deacon B, Lickel J, Abramowitz JS. Medical utilization across the anxiety disorders. *Journal of Anxiety Disorders* 2008;22(2):344-50. doi: 10.1016/j.janxdis.2007.03.004

5. Ialongo N, Edelsohn G, Werthamerlarsson L, et al. The significance of self-reported anxious symptoms in first-grade children - prediction to anxious symptoms and adaptive functioning in 5th grade. *Journal of Child Psychology and Psychiatry and Allied Disciplines* 1995;36(3):427-37. doi: 10.1111/j.1469-7610.1995.tb01300.x

6. Ollendick TH, March JS. *Phobic and anxiety disorders in children and adolescents: A clinician's guide to effective psychosocial and pharmacological interventions*. USA: Oxford University Press 2004.

7. Ollendick TH, King NJ, Muris P. Phobias in children and adolescents. In: Maj M, Akiskal HS, Lopez-Ibor JJ, et al., eds. Phobias New York: Wiley 2004:245-79.

8. Kendall PC, Hedtke KA. Cognitive-Behavioural Therapy for anxious children: Therapist manual. Ardmore: Workbook Publishing 2006.

For peer review only - http://bmjopen.bmj.com/site/about/guidelines.xhtml

9. Kendall PC, Robin JA, Hedtke KA, et al. Considering CBT with anxious youth? Think exposures. *Cognitive and Behavioral Practice* 2005;12(1):136-48. doi: 10.1016/s1077-7229(05)80048-3

10. Hofmann SG, Smits JAJ. Cognitive-behavioral therapy for adult anxiety disorders: A meta-analysis of randomized placebo-controlled trials. *Journal of Clinical Psychiatry* 2008;69(4):621-32.

11. Butler AC, Chapman JE, Forman EM, et al. The empirical status of cognitive-behavioral therapy: A review of meta-analyses. *Clinical Psychology Review* 2006;26(1):17-31. doi: 10.1016/j.cpr.2005.07.003

12. Hofmann SG, Asnaani A, Vonk IJJ, et al. The Efficacy of Cognitive Behavioral Therapy: A Review of Meta-analyses. *Cognitive Therapy and Research* 2012;36(5):427-40. doi: 10.1007/s10608-012-9476-1

13. Aschim B, Lundevall S, Martinsen EW, et al. General practitioners' experiences using cognitive behavioural therapy in general practice: A qualitative study. *Scandinavian Journal of Primary Health Care* 2011;29(3):176-80. doi: 10.3109/02813432.2011.595582

14. Wiebe E, Greiver M. Using cognitive behavioural therapy in practice: qualitative study of family physicians' experiences. *Canadian family physician Medecin de famille canadien* 2005;51:992-3.

15. Cavanagh K. Geographic Inequity in the Availability of Cognitive Behavioural Therapy in England and Wales: A 10-Year Update. *Behavioural and Cognitive Psychotherapy* 2014;42(4):497-501. doi: 10.1017/s1352465813000568

16. Shapiro DA, Cavanagh K, Lomas H. Geographic inequity in the availability of cognitive behavioural therapy in england and wales. *Behavioural and Cognitive Psychotherapy* 2003;31(2):185-92. doi: 10.1017/s1352465803002066

For peer review only - http://bmjopen.bmj.com/site/about/guidelines.xhtml

17. Öst LG. One-session treatment for specific phobias. *Behaviour Research and Therapy* 1989;27(1):1-7. doi: 10.1016/0005-7967(89)90113-7

18. Bower P, Gilbody S. Stepped care in psychological therapies: access, effectiveness and efficiency - Narrative literature review. *British Journal of Psychiatry* 2005;186:11-17. doi: 10.1192/bjp.186.1.11

19. National Collaborating Centre for Mental Health. Generalised anxiety disorder in adults: Management in primary, secondary and community care: British Psychological Society 2011.

20. Ollendick TH, Öst LG, Reuterskiöld L, et al. One-Session Treatment of Specific Phobias in Youth: A Randomized Clinical Trial in the United States and Sweden. *Journal of Consulting and Clinical Psychology* 2009;77(3):504-16. doi: 10.1037/a0015158

21. Öst LG, Svensson L, Hellstrom K, et al. One-session treatment of specific phobias in youths: A randomized clinical trial. *Journal of Consulting and Clinical Psychology* 2001;69(5):814-24. doi: 10.1037/0022-006x.69.5.814

22. Ryan SM, Strege MV, Oar EL, et al. One session treatment for specific phobias in children: Comorbid anxiety disorders and treatment outcome. *Journal of Behavior Therapy and Experimental Psychiatry* 2016;54:128-34.

23. Boutron I, Estellat C, Ravaud P. A review of blinding in randomized controlled trials found results inconsistent and questionable. *Journal of Clinical Epidemiology* 2005;58(12):1220-26. doi: 10.1016/j.clinepi.2005.04.006

24. Boutron I, Guittet L, Estellat C, et al. Reporting methods of blinding in randomized trials assessing nonpharmacological treatments. *Plos Medicine* 2007;4(2):370-80. doi: 10.1371/journal.pmed.0040061

For peer review only - http://bmjopen.bmj.com/site/about/guidelines.xhtml

25. Fergusson D, Glass KC, Waring D, et al. Turning a blind eye: the success of blinding reported in a random sample of randomised, placebo controlled trials. *British Medical Journal* 2004;328(7437):432-34. doi: 10.1136/bmj.37952.631667.EE

26. Sackett DL. Turning a blind eye - Why we don't test for blindness at the end of our trials. *British Medical Journal* 2004;328(7448):1136-36. doi: 10.1136/bmj.328.7448.1136-a

27. Ollendick TH, Halldorsdottir T, Fraire MG, et al. Specific phobias in youth: A randomized controlled trial comparing one-session treatment to a parent-augmented one-session treatment. *Behavior therapy* 2015;46(2):141-55.

28. Stallard P, Myles P, Branson A. The cognitive behaviour therapy scale for children and young people (CBTS-CYP): Development and psychometric properties. *Behavioural and cognitive psychotherapy* 2014;42(03):269-82.

29. Ollendick TH, Allen B, Benoit K, et al. The tripartite model of fear in children with specific phobias: Assessing concordance and discordance using the behavioral approach test. *Behaviour Research and Therapy* 2011;49(8):459-65. doi: 10.1016/j.brat.2011.04.003

30. Kindt M, Brosschot JF, Muris P. Spider phobia questionnaire for children (SPQ-C): A psychometric study and normative data. *Behaviour Research and Therapy* 1996;34(3):277-82. doi: 10.1016/0005-7967(95)00069-0

31. Boschen MJ, Veale D, Ellison N, et al. The emetophobia questionnaire (EmetQ-13): Psychometric validation of a measure of specific phobia of vomiting (emetophobia). *Journal of Anxiety Disorders* 2013;27(7):670-77. doi: 10.1016/j.janxdis.2013.08.004

32. Silverman WK, Albano AM. Anxiety Disorders Interview Schedule (ADIS-IV) Child and Parent Interview Schedules. Albany, NY: Greywind Publications 1996.

33. Silverman WK, Saavedra LM, Pina AA. Test-retest reliability of anxiety symptoms and diagnoses with the anxiety disorders interview schedule for DSM-IV: Child and

For peer review only - http://bmjopen.bmj.com/site/about/guidelines.xhtml

parent versions. *Journal of the American Academy of Child and Adolescent Psychiatry* 2001;40(8):937-44. doi: 10.1097/00004583-200108000-00016

34. Langley AK, Bergman RL, McCracken J, et al. Impairment in childhood anxiety disorders: Preliminary examination of the child anxiety impact scale-parent version. *Journal of Child and Adolescent Psychopharmacology* 2004;14(1):105-14. doi: 10.1089/104454604773840544

35. Chorpita BF, Yim L, Moffitt C, et al. Assessment of symptoms of DSM-IV anxiety and depression in children: A revised child anxiety and depression scale. *Behaviour research and therapy* 2000;38(8):835-55.

36. Chorpita BF, Moffitt CE, Gray J. Psychometric properties of the Revised Child Anxiety and Depression Scale in a clinical sample. *Behaviour research and therapy* 2005;43(3):309-22.

37. de Ross RL, Gullone E, Chorpita BF. The revised child anxiety and depression scale: a psychometric investigation with Australian youth. *Behaviour Change* 2002;19(02):90-101.

38. Bouvard M, Denis A. Reliability of the test-retest of the Revised Child Anxiety and Depression Scale (RCADS) and the assessment grid of the revised version of the Scale for Child Anxiety Related Emotional Disorders (SCARED-R). *L'Encéphale* 2012;38(6):524.

39. EuroQol Group. EuroQol-a new facility for the measurement of health-related quality of life. *Health policy (Amsterdam, Netherlands)* 1990;16(3):199-208.

40. Ravens-Sieberer U, Wille N, Badia X, et al. Feasibility, reliability, and validity of the EQ-5D-Y: results from a multinational study. *Quality of Life Research* 2010;19(6):887-97. doi: 10.1007/s11136-010-9649-x

For peer review only

41. Burstrom K, Bartonek A, Brostrom EW, et al. EQ-5D-Y as a health-related quality of life measure in children and adolescents with functional disability in Sweden: testing feasibility and validity. *Acta Paediatrica* 2014;103(4):426-35. doi: 10.1111/apa.12557

42. Ratcliffe J, Stevens K, Flynn T, et al. An assessment of the construct validity of the CHU9D in the Australian adolescent general population. *Quality of Life Research* 2012;21(4):717-25. doi: 10.1007/s11136-011-9971-y

43. Stevens K. Valuation of the Child Health Utility 9D Index. *Pharmacoeconomics* 2012;30(8):729-47.

44. Law D, Jacob J. Goals and Goal Based Outcomes (GBOs). 3rd ed. London: CAMHS Press 2013.

45. Marshall D, Wright B, Allgar V, et al. Social Stories in mainstream schools for children with autism spectrum disorder: a feasibility randomised controlled trial. *BMJ open* 2016;6(8):e011748.

46. Wright B, Tindall L, Littlewood E, et al. Computerised cognitive behaviour therapy for depression in adolescents: study protocol for a feasibility randomised controlled trial. Bmj Open 2014;4(10) doi: 10.1136/bmjopen-2014-00648847.

47. Wolitzky-Taylor KB, Horowitz JD, Powers MB, et al. Psychological approaches in the treatment of specific phobias: A meta-analysis. *Clinical Psychology Review* 2008;28(6):1021-37. doi: 10.1016/j.cpr.2008.02.007

48. Reynolds S, Wilson C, Austin J, et al. Effects of psychotherapy for anxiety in children and adolescents: A meta-analytic review. *Clinical Psychology Review* 2012;32(4):251-62. doi: 10.1016/j.cpr.2012.01.005

49. Jones B, Jarvis P, Lewis JA, et al. Trials to assess equivalence: The importance of rigorous methods. *British Medical Journal* 1996;313(7048):36-39.

For peer review only - http://bmjopen.bmj.com/site/about/guidelines.xhtml

50. Schulz KF, Altman DG, Moher D. CONSORT 2010 statement: updated guidelines for reporting parallel group randomized trials. *Annals of internal medicine* 2010;152(11):726-32.

51. Piaggio G, Elbourne DR, Pocock SJ, et al. Reporting of Noninferiority and Equivalence Randomized Trials Extension of the CONSORT 2010 Statement. *Jama-Journal of the American Medical Association* 2012;308(24):2594-604. doi: 10.1001/jama.2012.87802

52. Department of Health. Reference Costs Guidance 2015 to 2016. In: Health Do, ed., 2016:1 - 130.

53. Curtis L, Burns A. Unit Costs of Health and Social Care 2015. In: Unit PSSR, ed. Canterbury: University of Kent, 2015.

54. Royal Pharmaceutical Society of great Britain. British national formulary 72: Royal Pharmaceutical Society 2016.

55. Whitehead SJ, Ali S. Health outcomes in economic evaluation: the QALY and utilities. *British medical bulletin* 2010;96(1):5-21.

56. Manca A, Hawkins N, Sculpher MJ. Estimating mean QALYs in trial-based cost-effectiveness analysis: the importance of controlling for baseline utility. *Health Economics* 2005;14(5):487-96. doi: 10.1002/hec.944

57. Glick HA, Doshi JA, Sonnad SS, et al. Economic evaluation in clinical trials. Oxford: OUP 2014.

58. Hoch JS, Briggs AH, Willan AR. Something old, something new, something borrowed, something blue: a framework for the marriage of health econometrics and cost-effectiveness analysis. *Health Economics* 2002;11(5):415-30. doi: 10.1002/hec.678

For peer review only - http://bmjopen.bmj.com/site/about/guidelines.xhtml

59. Fenwick E, O'Brien BJ, Briggs A. Cost-effectiveness acceptability curves - facts, fallacies and frequently asked questions. *Health Economics* 2004;13(5):405-15. doi: 10.1002/hec.903

60. Lovell K, Bower p, Gellatly J, et al. Clinical effectiveness, cost-effectiveness and acceptability of low-intensity interventions in the management of obsessive-compulsive disorder: The obsessive-compulsive treatment efficacy randomised controlled trial (OCTET). *NIHR Journals Library* in press

61. Angell C, Alexander J, Hunt JA. 'Draw, write and tell': A literature review and methodological development on the 'draw and write' research method. *Journal of Early Childhood Research* 2015;13(1):17-28. doi: 10.1177/1476718x14538592

62. McWhirter J. The draw and write technique as a versatile tool for researching children's understanding of health and well-being. *International Journal of Health Promotion and Education* 2014;52(5):250-59.

63. Ritchie J, Spencer L, Bryman A, et al. Qualitative Data Analysis for Applied Policy Research. In: Bryman A, Burgess RG, eds. Analyzing Qualitative Data. London: Routledge 1994.

64. Dixon-Woods M. Using framework-based synthesis for conducting reviews of qualitative studies. *BMC medicine* 2011;9(1):1.

For peer review only

Reporting checklist for protocol of a clinical trial.

Based on the SPIRIT guidelines.

Instructions to authors

Complete this checklist by entering the page numbers from your manuscript where readers will find each of the items listed below.

Your article may not currently address all the items on the checklist. Please modify your text to include the missing information. If you are certain that an item does not apply, please write "n/a" and provide a short explanation.

Upload your completed checklist as an extra file when you submit to a journal.

In your methods section, say that you used the SPIRIT reporting guidelines, and cite them as:

Chan A-W, Tetzlaff JM, Altman DG, Laupacis A, Gøtzsche PC, Krleža-Jerić K, Hróbjartsson A, Mann H, Dickersin K, Berlin J, Doré C, Parulekar W, Summerskill W, Groves T, Schulz K, Sox H, Rockhold FW, Rennie D, Moher D. SPIRIT 2013 Statement: Defining standard protocol items for clinical trials. Ann Intern Med. 2013;158(3):200-207

		Reporting Item	Page Number
Title	#1	Descriptive title identifying the study design, population, interventions, and, if applicable, trial acronym	1
Trial registration	#2a	Trial identifier and registry name. If not yet registered, name of intended registry	2
Trial registration: data set	#2b	All items from the World Health Organization Trial Registration Data Set	2
Protocol version	#3	Date and version identifier	23
Funding	#4	Sources and types of financial, material, and other support	23
Roles and responsibilities: contributorship	#5a	Names, affiliations, and roles of protocol contributors	23
Roles and responsibilities:	#5b	Name and contact information for the trial sponsor	21

For peer review only

sponsor contact information			
Roles and responsibilities: sponsor and funder	#5c	Role of study sponsor and funders, if any, in study design; collection, management, analysis, and interpretation of data; writing of the report; and the decision to submit the report for publication, including whether they will have ultimate authority over any of these activities	21
Roles and responsibilities: committees	#5d	Composition, roles, and responsibilities of the coordinating centre, steering committee, endpoint adjudication committee, data management team, and other individuals or groups overseeing the trial, if applicable (see Item 21a for data monitoring committee)	21
Background and rationale	#6a	Description of research question and justification for undertaking the trial, including summary of relevant studies (published and unpublished) examining benefits and harms for each intervention	5
Background and rationale: choice of comparators	#6b	Explanation for choice of comparators	10
Objectives	#7	Specific objectives or hypotheses	6
Trial design	#8	Description of trial design including type of trial (eg, parallel group, crossover, factorial, single group), allocation ratio, and framework (eg, superiority, equivalence, non-inferiority, exploratory)	7
Study setting	#9	Description of study settings (eg, community clinic, academic hospital) and list of countries where data will be collected. Reference to where list of study sites can be obtained	7
Eligibility criteria	#10	Inclusion and exclusion criteria for participants. If applicable, eligibility criteria for study centres and individuals who will perform the interventions (eg, surgeons, psychotherapists)	6
Interventions: description	#11a	Interventions for each group with sufficient detail to allow replication, including how and when they will be administered	8

For peer review only

Interventions: modifications	#11b	Criteria for discontinuing or modifying allocated interventions for a given trial participant (eg, drug dose change in response to harms, participant request, or improving / worsening disease)	See note 1
Interventions: adherance	#11c	Strategies to improve adherence to intervention protocols, and any procedures for monitoring adherence (eg, drug tablet return; laboratory tests)	10
Interventions: concomitant care	#11d	Relevant concomitant care and interventions that are permitted or prohibited during the trial	N/A
Outcomes	#12	Primary, secondary, and other outcomes, including the specific measurement variable (eg, systolic blood pressure), analysis metric (eg, change from baseline, final value, time to event), method of aggregation (eg, median, proportion), and time point for each outcome. Explanation of the clinical relevance of chosen efficacy and harm outcomes is strongly recommended	9
Participant timeline	#13	Time schedule of enrolment, interventions (including any run-ins and washouts), assessments, and visits for participants. A schematic diagram is highly recommended (see Figure)	N/A
Sample size	#14	Estimated number of participants needed to achieve study objectives and how it was determined, including clinical and statistical assumptions supporting any sample size calculations	14
Recruitment	#15	Strategies for achieving adequate participant enrolment to reach target sample size	7
Allocation: sequence generation	#16a	Method of generating the allocation sequence (eg, computer-generated random numbers), and list of any factors for stratification. To reduce predictability of a random sequence, details of any planned restriction (eg, blocking) should be provided in a separate document that is unavailable to those who enrol participants or assign interventions	7
Allocation concealment	#16b	Mechanism of implementing the allocation sequence (eg, central telephone; sequentially numbered, opaque, sealed	8

For peer review only

mechanism		envelopes), describing any steps to conceal the sequence until interventions are assigned	
Allocation: implementation	#16c	Who will generate the allocation sequence, who will enrol participants, and who will assign participants to interventions	7
Blinding (masking)	#17a	Who will be blinded after assignment to interventions (eg, trial participants, care providers, outcome assessors, data analysts), and how	8
Blinding (masking): emergency unblinding	#17b	If blinded, circumstances under which unblinding is permissible, and procedure for revealing a participant's allocated intervention during the trial	8
Data collection plan	#18a	Plans for assessment and collection of outcome, baseline, and other trial data, including any related processes to promote data quality (eg, duplicate measurements, training of assessors) and a description of study instruments (eg, questionnaires, laboratory tests) along with their reliability and validity, if known. Reference to where data collection forms can be found, if not in the protocol	11
Data collection plan: retention	#18b	Plans to promote participant retention and complete follow-up, including list of any outcome data to be collected for participants who discontinue or deviate from intervention protocols	16
Data management	#19	Plans for data entry, coding, security, and storage, including any related processes to promote data quality (eg, double data entry; range checks for data values). Reference to where details of data management procedures can be found, if not in the protocol	21
Statistics: outcomes	#20a	Statistical methods for analysing primary and secondary outcomes. Reference to where other details of the statistical analysis plan can be found, if not in the protocol	15
Statistics: additional analyses	#20b	Methods for any additional analyses (eg, subgroup and adjusted analyses)	15
Statistics: analysis population and missing data	#20c	Definition of analysis population relating to protocol non-adherence (eg, as randomised analysis), and any statistical methods to handle missing data (eg, multiple imputation)	15

For peer review only

Data monitoring: formal committee	#21a	Composition of data monitoring committee (DMC); summary of its role and reporting structure; statement of whether it is independent from the sponsor and competing interests; and reference to where further details about its charter can be found, if not in the protocol. Alternatively, an explanation of why a DMC is not needed	21
Data monitoring: interim analysis	#21b	Description of any interim analyses and stopping guidelines, including who will have access to these interim results and make the final decision to terminate the trial	21
Harms	#22	Plans for collecting, assessing, reporting, and managing solicited and spontaneously reported adverse events and other unintended effects of trial interventions or trial conduct	22
Auditing	#23	Frequency and procedures for auditing trial conduct, if any, and whether the process will be independent from investigators and the sponsor	21
Research ethics approval	#24	Plans for seeking research ethics committee / institutional review board (REC / IRB) approval	22
Protocol amendments	#25	Plans for communicating important protocol modifications (eg, changes to eligibility criteria, outcomes, analyses) to relevant parties (eg, investigators, REC / IRBs, trial participants, trial registries, journals, regulators)	21
Consent or assent	#26a	Who will obtain informed consent or assent from potential trial participants or authorised surrogates, and how (see Item 32)	20
Consent or assent: ancillary studies	#26b	Additional consent provisions for collection and use of participant data and biological specimens in ancillary studies, if applicable	N/A
Confidentiality	#27	How personal information about potential and enrolled participants will be collected, shared, and maintained in order to protect confidentiality before, during, and after the trial	21
Declaration of interests	#28	Financial and other competing interests for principal investigators for the overall trial and each study site	23
Data access	#29	Statement of who will have access to the final trial dataset,	21

For peer review only

		and disclosure of contractual agreements that limit such access for investigators	
Ancillary and post trial care	#30	Provisions, if any, for ancillary and post-trial care, and for compensation to those who suffer harm from trial participation	N/A none
Dissemination policy: trial results	#31a	Plans for investigators and sponsor to communicate trial results to participants, healthcare professionals, the public, and other relevant groups (eg, via publication, reporting in results databases, or other data sharing arrangements), including any publication restrictions	22
Dissemination policy: authorship	#31b	Authorship eligibility guidelines and any intended use of professional writers	22
Dissemination policy: reproducible research	#31c	Plans, if any, for granting public access to the full protocol, participant-level dataset, and statistical code	N/A none
Informed consent materials	#32	Model consent form and other related documentation given to participants and authorised surrogates	20
Biological specimens	#33	Plans for collection, laboratory evaluation, and storage of biological specimens for genetic or molecular analysis in the current trial and for future use in ancillary studies, if applicable	N/A

Author notes

1. N/A - no moficiations

The SPIRIT checklist is distributed under the terms of the Creative Commons Attribution License CC-BY-ND 3.0. This checklist was completed on 26. June 2018 using http://www.goodreports.org/, a tool made by the EQUATOR Network in collaboration with Penelope.ai

For peer review only
