# Supplementary material for: Clinical and cost-effectiveness of one-session treatment (OST) versus multisession cognitive–behavioural therapy (CBT) for specific phobias in children: protocol for a non-inferiority randomised controlled trial
Source: BMJ Open. 2018 Aug 17;8(8):e025031. doi: 10.1136/bmjopen-2018-025031 (PMC6104754; doi:10.1136/bmjopen-2018-025031)
Supplement: Reviewer comments [file bmjopen-2018-025031.reviewer_comments.pdf]

PEER REVIEW HISTORY

BMJ Open publishes all reviews undertaken for accepted manuscripts. Reviewers are asked to complete a checklist review form (**http://bmjopen.bmj.com/site/about/resources/checklist.pdf**) and are provided with free text boxes to elaborate on their assessment. These free text comments are reproduced below.

ARTICLE DETAILS

**TITLE (PROVISIONAL)**	Clinical and Cost-effectiveness of One Session Treatment (OST) vs. Multi-session Cognitive Behaviour Therapy (CBT) for Specific Phobias in Children: Protocol for a Non-inferiority Randomised Controlled Trial
**AUTHORS**	Wright, Barry; Cooper, Cindy; Scott, Alex; tindall, Lucy; Ali, Shehzad; Bee, Penny; Biggs, Katie; Breckman, Trilby; Davis III, Thompson; Gega, L; Hargate, Rebecca; Lee, Ellen; Lovell, Karina; Marshall, David; McMillan, Dean; Teare, M; Wilson, Jon

This article was not externally reviewed at BMJ Open. Protocols that have been independently assessed prior to submission to BMJ Open are usually fast-tracked to publication on the grounds that further substantial changes will not be possible. This independent assessment will usually be external, independent review for both a substantial grant award from a non-commercial or government funder and ethics approval.
